# Decoding sebaceous gland functions and diseases: insights from domestic animals

**DOI:** 10.1007/s11259-026-11128-0

**Published:** 2026-03-13

**Authors:** Helga Pfannkuche, Juliane Maus, Kathrin M. Engel, Jürgen Schiller, Marlon R. Schneider

**Affiliations:** 1 Institute of Veterinary Physiology, Leipzig University, An den Tierkliniken 7, 04103 Leipzig, Germany; 2 Institute of Medical Physics and Biophysics, Faculty of Medicine, Leipzig University, Härtelstr. 16-18, 04107 Leipzig, Germany

**Keywords:** Sebaceous glands, Meibomian glands, Sebum, Skin physiology, Domestic animals

## Abstract

Skin sebaceous glands (SGs) synthesize and secrete sebum, a mixture of lipids and cellular debris that defends the external body surface against physicochemical challenges. Recent data define the SG as a dynamic entity with potential functions beyond skin protection, including immunomodulatory actions and the regulation of energy metabolism. We postulate that the SG also has important, unrecognized roles in physiological and pathological processes in domestic animals. Conversely, data derived from domestic animals may have translational relevance for humans. This review article summarizes SG structural and functional features in the most widespread species domesticated for food (cattle, sheep, goats, and pigs), work (horses), and companionship (dogs and cats). Our survey reveals hitherto unrecognized roles of the SG in diverse pathophysiological processes. Among other fascinating facts, we learn that sebum has an exquisite and unique lipid composition in each of the considered species. Furthermore, sebum is essential for e.g., wool production but also a carrier for the most important cat protein causing allergic reactions in humans, and dogs and cats may develop a SG-related skin illness resembling acne that is potentially relevant as a model for the human disease. This critical review provides a foundation for future interdisciplinary studies in a largely neglected area with great potential for advancing animal welfare and human health.

## Introduction

**Fig. 1:**
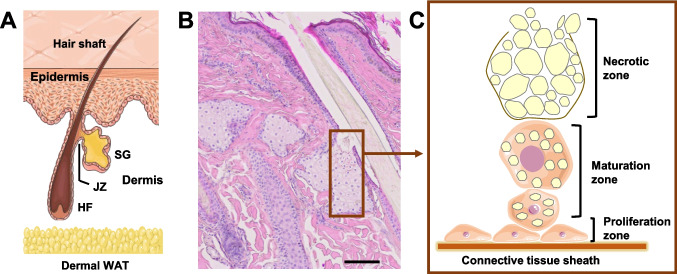
Microscopic anatomy and physiology of sebaceous glands. **A**: General anatomy of the pilosebaceous unit, including the hair follicle (HF) and an associated sebaceous gland (SG). The junctional zone (JZ) and the dermal white adipose tissue (WAT) are indicated. **B:** Histological appearance of an adult horse forehead SG stained with hematoxylin & eosin. The scale is equivalent to 100 µm. **C:** SG homeostasis is based on the proliferation of progenitor cells in the proliferation zone, the stochastic selection of individual cells that start synthesizing lipids and move into the maturation one, and their death coupled with secretion of lipids and cellular debris into the HF canal. **A** and **C** were drawn by using a picture from Servier Medical Art (licensed under a Creative Commons Attribution 3.0 Unported License; https://creativecommons.org/licenses/by/3.0/). The original drawing in **A** depicted human skin

The mammalian skin performs a multitude of essential functions, serving as a physical, chemical, and biological barrier against the external environment, while also acting as a sensory and endocrine organ. Structurally, it comprises the epidermis—a continuously renewing stratified epithelium—and the underlying dermis, which contains skin appendages such as hair follicles, sebaceous glands (SGs), and sweat glands, embedded within a fibroblast-rich stromal matrix (Fig. 1A). SGs are exocrine glands associated with hair follicles, and their lipid-rich secretion, known as sebum, primarily functions to lubricate and protect both the skin surface and hair shafts (Smith and Thiboutot 2008; Schneider and Paus 2010). The composition of sebum lipids varies significantly across mammalian species, likely reflecting differences in phylogeny and ecological demands (Nicolaides 1974; Vanderwolf et al. 2023). In fur-bearing animals, sebum plays a critical role in functions such as waterproofing (Dahlhoff et al. 2016), whereas in humans, its precise physiological role remains a subject of ongoing debate (Zung and McBride 2025). Dysregulation of sebum secretion, including changes both in the amount of sebum and in the relative amount of its lipid classes, represents a central pathogenic mechanism in acne vulgaris—the most prevalent dermatological disorder globally and a significant health burden during adolescence (van Steensel 2019). Moreover, aberrant sebaceous gland activity has been implicated in other chronic inflammatory skin diseases, including atopic dermatitis and psoriasis (Zouboulis et al. 2016).

Importantly, sebaceous glands should not be regarded merely as lipid-producing structures. Instead, they are highly dynamic and responsive to both local and systemic cues (Schmidt et al. 2025). As such, SGs offer a valuable model for investigating broader biological processes, encompassing cell adhesion and metabolism (Sipilä et al. 2022), organ size regulation (Yosefzon et al. 2018), lipid biosynthesis and metabolism (Exner et al. 2019), host–microbiome interactions (Kobayashi et al. 2019), and systemic lipid and energy homeostasis (Choa et al. 2021).

### Developmental and structural aspects of the sebaceous gland

SGs are hair follicle (HF)-associated exocrine skin glands found in the dermis of most mammals, notable exceptions being cetaceans and hippos (Springer et al. 2021). The general SG microscopic structure (Fig. 1A,B) is quite similar in most mammal species (Schneider and Paus 2010; Hinde et al. 2013). The glands can be uni-, bi-, or multilobular and consist of secretory acini that are connected to the distal portion of the HF via a keratinized duct. Together with the arrector pili muscle, the SG and the HF form the pilosebaceous unit. The region where the SG duct inserts into the pilar canal is termed the junctional zone, and the HF region distal to it, where the hair shaft separates from the follicular epithelium, is known as the infundibulum. Sebum, the SG product, travels via the duct into the HF canal and eventually reaches the skin surface. The gland is surrounded by a collagen-rich connective tissue sheath that generates a trabecular system separating the individual acini from each other and from the dermis. This sheath is lined by small, mitotically active glandular cells forming the proliferation zone (Fig. 1C) (Schneider and Paus 2010; Yaba et al. 2024). Some of these cells exit this proliferative pool and engage into sebaceous differentiation: they massively accumulate cytoplasmic lipid droplets, lose other subcellular structures, and are displaced towards the center of the gland, forming the maturation zone (Schmidt et al. 2024). As they approach the duct, the terminally differentiated sebocytes disintegrate and form the sebum, which consists of a complex mixture of lipids and cellular debris. This process is termed holocrine secretion, and occurs at the (rather misnamed) necrotic zone.

SG development, renewal, and regeneration has been described in quite detail in laboratory mice. In this species, the first sebocytes emerge in the late fetal/early postnatal period. Prior to the appearance of sebocytes, a discrete population of cells that express both leucine-rich repeats and immunoglobulin-like domains protein 1 (Lrig1) and SRY-box transcription factor 9 (Sox9) forms within the developing hair peg. As development proceeds, lineage-restricted progenitor cells expressing only Lrig1 separate, allocate to the junctional zone, and generate the first sebocytes (Frances and Niemann 2012). These Lrig1-positive cells simultaneously expand to a population of equipotent progenitor cells that renew the SG under homeostatic conditions (Frances and Niemann 2012; Andersen et al. 2019). Human SGs are active in utero, and contribute, at least in part, to the formation of the vernix caseosa (Rissmann et al. 2006).

In addition to those associated with the HF, there are so-called "free” or specialized SGs. They release their secretions directly onto the surface of the skin and can be divided into two groups: glands at mucocutaneous junctions and pheromone-producing glands. The first type is usually located at the body orifices and mainly fulfils antimicrobial functions. SGs of this type are located at the edges of the lips (Fordyce glands), at the areola (Montgomery glands), and in the anogenital region (Tyson glands) (Schneider and Paus 2010). The Meibomian glands, localized in the eyelids (also called tarsal glands) also belong to this group of free SGs. Their secretion (meibum) supports the formation and stability of the tear film by preventing evaporation of its aqueous component (Dahlhoff et al. 2016; Butovich 2017). The second group comprises large aggregates of SG tissue that occur in specific regions of the body in some species and release varying amounts of pheromones depending on reproductive activity. These glands include, for example, the preputial glands of various rodents (Brouette-Lahlou et al. 1991), the costovertebral gland of the hamster (Takayasu and Adachi 1970) and the metatarsal gland of the European roe deer (Wood 2003).

### Overview of sebum composition across species

**Fig. 2:**
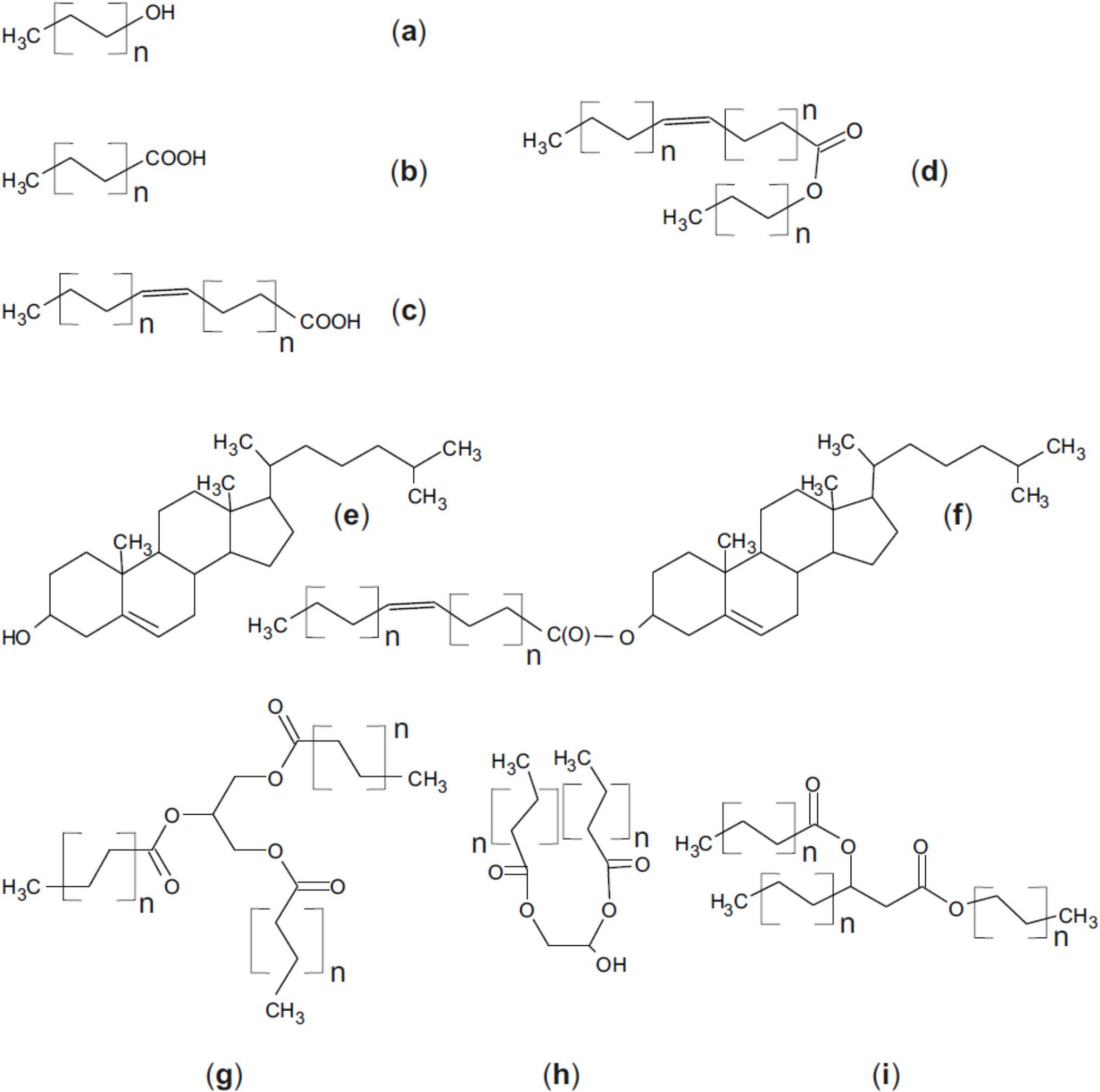
Overview of the apolar lipid classes mentioned in the text of this review. All the more complex lipids consist of fatty alcohols (**A**) or saturated (**B**) or unsaturated (**C**) fatty acids. Under conditions of an ester condensation simple wax esters (**D**) are generated. Another important component is cholesterol, either as free compound (**E**) or esterified with a fatty acid (**F**). Finally, significant amounts of triacylgylcerols (**G**) and waxes are found. Wax diesters are further divided into type 1 (**H**) and type 2 (**I**). Type 1 wax diesters are formed from wax monoesters whose FA is an α-hydroxy fatty acid. The presence of the hydroxy group allows esterification with a second FA to form a diester. To form a type 2 wax diester, a wax monoester is required whose alcohol moiety is an alkanediol. One FA can be esterified to each hydroxyl group. In type 2a wax monoesters, the alkanediol is a 1,2-diol. Note that the length of the acyl residues is usually a multiple of 2, i.e. the number of carbon atoms is even numbered. Odd numbered acyl chains are usually less common. Natural fatty acids normally possess 16 or even more carbon atoms

Sebum lipids of all species analyzed so far are largely apolar. This differentiates them from those of epidermal origin, which are mainly polar. Epidermal lipids are produced by keratinocytes and fill their intercellular spaces; they are found only to a small extent on the hair or skin surface, and include ceramides, free fatty acids (FFA) and cholesterol. The main lipid classes found in sebum are cholesterol, wax esters (WE), squalene, triacylglycerols (TAG), and FFAs (Fig. 2) (Smith and Thiboutot 2008). Epidermal lipids have a rather consistent composition across mammalian species, while the composition of sebum shows large species-specific differences (Nicolaides et al. 1968; Thalheim and Schneider 2024).

In addition to cholesterol, sterols as lathosterol, desmosterol, lanosterol, dihydrolanosterol, and agnosterol are often present in the form of steryl esters, i.e., esterified with stearic acid. Furthermore, the sebum of many species contains large quantities of WE, a lipid type not found in any other tissue or secretion of mammals. WEs consist of an alcohol that is esterified with unsaturated (e.g., oleic acid (C18:1), linoleic acid (C18:2)) or saturated FAs (e.g., palmitic acid (C16:0), stearic acid (C18:0)). The required branched-chain FAs are synthesized from the branched amino acids valine and isoleucine (Pappas 2015). WEs can be divided into wax monoesters, wax diesters and wax triesters, depending on the number of esterified FAs. Wax diesters are further divided into type 1 and type 2. Type 1 wax diesters are formed from wax monoesters whose FA is an α-hydroxy FA. The presence of the hydroxy group enables esterification with a second FA, resulting in a diester. To form a type 2 wax diester, a wax monoester is required, the alcohol moiety of which is an alkanediol. FAs can be esterified to each hydroxyl group. In type 2a wax monoesters, the alkanediol is a 1,2-diol. If one of the FAs is a hydroxy acid, this can be esterified with a third FA to form a wax triester. The wax diesters type 2b contain an alkanediol in the form of a 2,3-diol and have so far been detected in the uropygial gland of birds and on the skin surface of macaques (*Macaca fascicularis*) (Nishimaki-Mogami et al. 1988). WEs are the most hydrophobic sebum components and, therefore, contribute most effectively to preventing dehydration and soaking of the skin. Due to their microstructure, they also develop the so-called lotus effect in combination with water, which removes dirt from the skin (Pappas 2015).

Squalene, only an intermediate substrate in cholesterol biosynthesis in almost all animal cells, atypically accumulates in sebum (Nicolaides 1974). It is a long, unsaturated hydrocarbon made up of several isoprene units with high hydrophobicity and fluidity (Pappas 2015). Human sebum also contains TAGs, and sapienic acid (cis-6-hexadecenoic acid, C16:1) makes up the largest proportion of their FAs. The basis for its synthesis is C16:0, which is formed in sebocytes from C18:2, and it is supposed to have high antimicrobial activity, with a particularly selective effect against *Staphylococcus aureus* (Smith and Thiboutot 2008; Pappas 2015). In addition to TAG, WE and squalene, FFAs as well as mono- and diglycerides may also be found in sebum samples. The latter are presumably formed from the TAG of the sebum when these are hydrolyzed by bacterial lipases (Freinkel and Shen 1969).

The species-specific composition of the sebum will be described in detail in the chapters on the respective animal species, a comparative overview is provided in Table 1.

**Table 1:** Overview of sebum composition in domestic animals (Nicolaides et al. 1968; Wheatly, 1986) in comparison to humans and mice (Smith and Thiboutot 2008). Components were identified by thin layer chromatography and subsequently quantified. For pigs and goats there is no quantitative information available. The sample source was sebum isolated from the skin surface (humans), from hairs (mice) or from elution of lipids from hair and skin (domestic animals)

	Wax esters	Sterol esters	Sterols	FFA	TAG	Squalene	Lactones
Human	26%		Cholesterol (2%)	Triglycerides, diglycerides and free fatty acids (57%)		12%	
Mouse	5%		Cholesterol (13%)	Triglycerides, diglycerides and free fatty acids (9%)			
Cattle	Wax diester type 1 (38%) wax diesters type 2a (8%) wax triesters (30%) lyso derivates of wax esters (2%)	Cholesteryl esters (3%)	Free cholesterol (4%)	FFA (2.3%) Free fatty alcohols (0.6%)	TAG (3.6%)		
Sheep	Wax monoesters (10%) wax diester type 1 (5%) wax diesters type 2a (4%)	Steryl monoesters (46%); steryl diesters (12%);	Free sterols (12%)				
Goat	(x)	x	x	(x)			
Pig		x	x	x	x		
Horse		Steryl monoesters (38%)	Free sterols (14%)		(x)		48%
Cat	Wax diester type 1 (66%)		Free sterols (6%)				
Dog	Wax diesters type 2a (35%)	Steryl monoesters (42%)	Free sterols (9%)				

x: detected but not quantified, (x): presumed

### Objectives and study methodology

This article summarizes the available information about structural, functional, and pathological features of SGs in the most common domestic mammals. It sets itself apart from a previous, excellent survey of functional aspects of sebum in animals (Vanderwolf et al. 2023) by including more detailed structural and disease-related aspects and by focusing on a selected number of domestic animal species. Besides offering a comprehensive resource regarding SG and sebum functions in these species for the first time, we aim at identifying similarities and key divergences compared to humans and mice, the most employed experimental animal models in SG research. It has been shown that metabolic disorders in humans aggravate skin lipid abnormalities by influencing SG activity. Insulin resistance, for instance, increases the ratio of saturated to unsaturated fatty acids, favoring colonization by *S. aureus* over *C. acnes*, and commensal. *S. aureus*-derived lipases degrade lipids into pro-inflammatory FFAs, thus aggravating keratinocyte dysfunction and inflammation (Kreouzi et al. 2025). We anticipate that similar processes may lead to the development of skin diseases in domestic animals. Conversely, studying SG pathophysiology in these species may be instructive for understanding manifold processes relevant for human health.

**Fig. 3:**
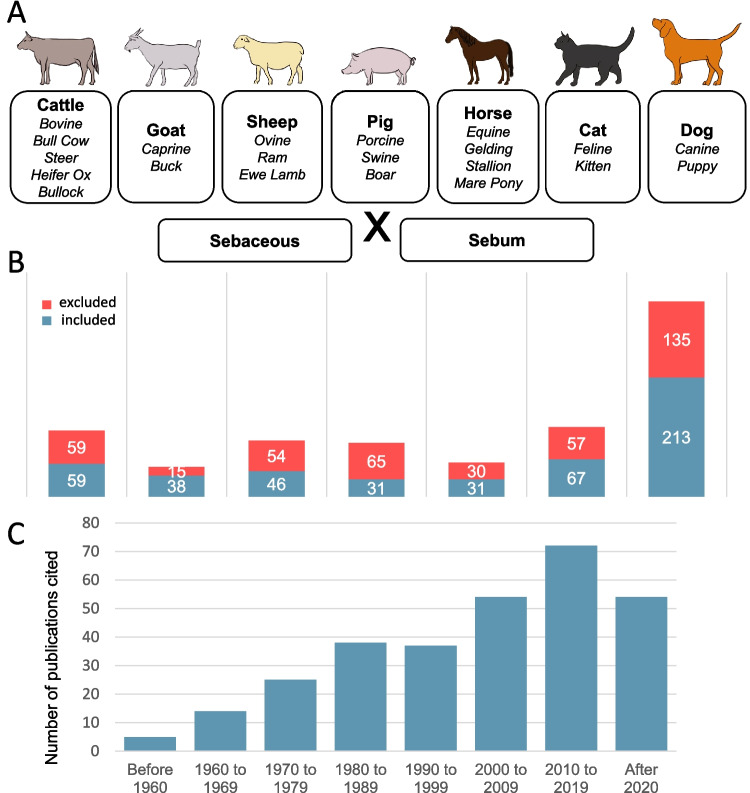
**A**: Species-specific terms and search string terms (Sebaceous or Sebum) used to identify relevant literature in PUBMED and Google Scholar. **B**: Number of the original publications included in the further analysis and of those excluded, broken down to each species. **C**: Number of original publications included in the review distributed according to decade of their publication

To identify relevant literature, we searched NCBI PUBMED and Google Scholar using the search string ("sebum" OR "sebaceous") AND species-specific terms. Here, we include original reports published up to October 2025. For the PUBMED search, similar terms were covered by Medical Subject Heading (MeSH) terms; these were included manually for the Google Scholar search (Fig. 3A). Results from Google Scholar were considered only if search terms appeared in the title of publications. Textbooks were considered to a smaller extent. Most of these textbooks focused on dermatology, but the topics skin lipids, propaedeutics, histopathology, general pathology, specialized pathology and anatomy were also included.

In total, 859 publications were found in NCBI PUBMED and Google scholar, and 41 publications were identified in textbooks. Of these 900 publications, 485 were included and 415 were not considered. Publications that contained information on the structure, function and control of the SGs as well as the sebum composition, pathologies and SG-related characteristics of the respective species were taken into account. Publications containing general information on the structure, function and control of SGs in other species were also included. Publications dealing with other skin-related issues, only listing SGs as an existing structure, or solely focusing on the therapy of SG-associated diseases were excluded. Figure 3B shows the total number of publications retrieved as well as the number of retained/excluded publications broken down to each species. Importantly, while we assessed in detail each one of the 485 retained publications, not all of them are cited within the text and in the reference list, as some of them simply reported a known pathology or, in general, did not provide any additional information. Figure 3C shows the distribution of the included publications to the decade of their publication. Notably, while a substantial proportion of these reports were published before the year 2000, the growing number of studies published after 2020 indicates a rising interest in this topic.

## Sebaceous gland and sebum function in animals domesticated for food

### Cattle

#### Structure of the gland

In cattle (*Bos taurus*), the anlage of the SGs are formed between the 119th and 134th day of foetal development (Lyne and J Heideman 1959). As usual, SGs are associated with HFs, except for those at the outer ear canal and on the eyelids (Sinha 1964; Smith 1975; Rohankar et al. 2018). The peripheral layer of the gland makes up an average of 11%, the maturing cells make up 57%, and the degenerating cells 32% of the SG volume (Jenkinson et al. 1985). Areas on the body surface lacking SGs include the planum nasale, teats, horns and claws (Sinha 1964; Naik et al. 2024). Particularly large SG aggregates are located at mucocutaneous junctions and at junctions of skin and horn (Sinha 1964; Blazquez et al. 1987a). The largest SGs are located on the abdomen, in the axillary region, the inguinal region and on the udder, although the latter are not very numerous, as the hair density in this region is low (Sinha 1964; Ludewig et al. 1996; Naik et al. 2024). For a comparison between species see Table 2. Some authors assume an inverse proportionality between the SG size and the thickness of the associated hair (Ludewig et al. 1996). The highest SG density is found on the vulva and in the perineal region of cows, in particular during estrus (Sinha 1964; Blazquez et al. 1987a). These SGs consist of several lobules, whereas those on the rest of the body are generally bilobular in structure. In cows, SGs located on vulva and perineum are presumably responsible for the olfactory interest of bulls in the perineal region in proestrus, although the exact source of the odor, and in particular whether it results from a modification of sebum by bacteria, remains unknown. Male cattle also have large SGs in the perineal region, but their volume is significantly smaller than in females (Blazquez et al. 1987a). High percentages of sebaceous glands and apocrine sweat glands per unit area were found in the tail root region of Holstein cows (Fujii et al. 2024). The high density of sebaceous and apocrine sweat glands in this skin region is observed in many other ungulates, not only in cattle.

**Table 2:** Overview of regions with high sebum production as well as specialized sebaceous glands in domestic animals as described so far. Meibomian glands are not included as they have been described in all mentioned species (except for goat). For detailed literature please refer to the main text

Cattle	High production	Vulva, perineum, mucocutaneous transitions, transitions of skin and horn, abdomen, axillary region, inguinal region, udder
Sheep	High production	Dorsal neck, lips, vulva, anus, perineum, larger sebaceous glands in hairy regions than in woolly regions, udder
	Specialized glands	Infraorbital gland, interdigital gland, inguinal gland only in female sheep
Goat	High production	dorsal neck, lateral abdomen, snout, scrotum, inner sides of limbs, ventral side of auricles
	Specialized glands	Intercornual gland, subcaudal gland, mental gland (only in bucks) preputial gland (only in bucks), periareolar glands in teat skin
Pig	High production	Inner side of the pinna
Horse	High production	Eyelids, teats, mane ridge, submandibular region, coronet band, mucocutaneous transitions
Cat	High production	mucocutaneous junctions, interdigital, dorsal neck and trunk, chin, dorsal root of the tail
	Specialized glands	Mental organ, circumoral organ, glandulae tori between the toes
Dog	High production	mucocutaneous junctions, interdigital, dorsal neck and trunk
	Specialized glands	dorsal caudal gland (rudimentary), parts of the circumanal gland, preputial gland in African wild dogs

The Meibomian gland of cattle, in contrast to that of humans, has a single excretory duct that is up to 1 mm long. No glandular acini are located along this duct, instead, the terminal end splits into multiple smaller ducts that lead to the glandular lobules (Jester et al. 1981). Of note, buffalo SGs are larger and consist of more lobules than those of cattle, while buffalo sweat glands are less developed. This might be interpreted as sebum having an evaporation-inhibiting effect on sweat and, thus, impairing thermoregulation (Ibrahim and Hussin 2017).

After removal of the skin lipids, the amount of sebum on the skin of cattle increased continuously over the first 24 h, with the most marked increase within the first 18 h (Smith 1975). This time seems therefore to be required to restore the protective lipid film of the skin.

#### Composition of the sebum

Within the Bovidae family, the composition of skin lipids is quite homogeneous, only the proportions of the constituents vary slightly between species (Lindholm et al. 1981). A comparative view on sebum composition is given in Table 1. The lipid composition of the skin surface of cows was determined by quantitative thin layer chromatography (Downing and Lindholm 1982). According to these data, cow sebum consists of 37.9% type 1 wax diesters, 29.9% wax triesters, 7.9% type 2 wax diesters, 4.0% free cholesterol, 3.6% TAGs, and 3.0% cholesteryl esters. In addition, 2.3% FFAs and 0.6% FF alcohols are present, as well as 8.5% lyso-derivatives of wax diesters and wax triester, which result from the hydrolysis of an acyl group. The remaining lipids could not be identified.

Several studies compared the lipids extracted directly from the SGs of cattle with those from the skin surface. In 12-month-old Ayshire bulls sampled in the winter, only slight differences in the composition of the lipids were found by thin layer chromatography. The lipids within the SG contained higher levels of phospholipids (PL) than the surface samples, particularly PL containing C16:0 or C18:0. In addition, the sebum contained higher levels of non-esterified FA and lower levels of TAG compared to the surface lipids (Smith and Ahmed 1976). A similar study on Holstein Friesian heifers found a higher content of free cholesterol and PL and fewercholesteryl esters in the surface lipids than in the samples isolated directly from the glands. This might be explained by the cleavage of cholesteryl esters by bacterial esterases on the skin surface (McMaster et al. 1985b). Both studies supported the assumption that the skin surface lipids in cattle consist mainly of sebum. The FAs of the TAG fraction consisted of 33% C16:0, 19% C18:1, 15% myristic acid (C14:0), 13.4% C18:0, 9.2% C18:2 and 5.9% palmitoleic acid (C16:1) and 4.5% not classified FA (McMaster et al. 1985b). Epidermal TAGs have only a very low content of C18:2, making the C18:2-rich TAG a suitable marker for sebum lipids (McMaster et al. 1985b). Accordingly, when SGs isolated from Holstein Friesian heifers were provided with radioactively labelled C16:0, C18:1 and C18:2, C18:2 was incorporated into the TAGs at a higher proportion than the other FAs (McMaster et al. 1985a). Additionally, it was observed that 75% of the FAs provided were incorporated into TAG and PL. Although non-esterified FAs are present in the plasma of cattle with high metabolic turnover, blood lipids do not seem be a significant source of sebum lipids. This is based on the observation that these FAs had a low proportion of C18:2. However, relevant amounts of phosphorylcholine were found in the blood serum of bulls, which occurs as a component of some PL (such as phosphatidylcholine), and sphingomyelins. Consequently, it cannot be excluded that C18:2 is present in the blood serum of cattle, but is bound to PL and has therefore not been recorded in previous analyses (Jakop et al. 2022). Such a binding might be confirmed by digestion of serum with phospholipases and evaluation of the released C18:2. The excretion of radioactively labelled lipids reached its maximum four days after injection of the radioactive FAs, and a second peak appeared after eight to nine days. This was interpreted as a cyclical activity of the SG due to a phasic development of the sebocytes (McMaster et al. 1986).

Only recently, analyses of the tissue expression of diacylglycerol acyltransferases (DGAT), enzymes that convert diacylglycerol and fatty acyl-CoA into TAG, showed that the genes for DGAT2L3/AWAT1, DGAT2L4/AWAT2, and DGAT2L6 are highly expressed in cattle sebum and sebocytes, revealing enrichment for TAG metabolic processes and their biosynthesis (Esfahani et al. 2025).

As expected, the secretion of the cattle Meibomian gland differs from that of skin SGs. Bovine meibum includes WEs (35%), sterol esters (30%), FFAs (5%), TAGs (2%), and unidentifiable lipids (25%) (Nicolaides et al. 1984).

In cows, a substance in the teat called lactosebum (also known as keratin plug), protects the teat canal from the invasion of bacteria. As it only has a lipid content of 7% (Lojda et al. 1974), and as there is no evidence of SGs on the teats (Sinha 1964), assigning this substance to sebum is questionable.

#### Influences on sebum secretion

##### Hormonal regulation

Sebum production in cattle seems not to be controlled by testosterone. This is based on the lack of correlation between plasma testosterone levels and the amount of produced sebum in cattle (O'Kelly 1985). Moreover, neither the amount of sebum nor its composition differed between bulls and heifers. Castration of neonatal or adult males as well as treatment of bulls with testosterone had also no significant effect on sebum quantity and composition (O'Kelly 1985). These surprising findings are difficult to reconcile with the established impact of testosterone on sebum production in numerous other mammals. Influences on sebum production and composition are also listed in Table 3.

**Table 3:** Physiological influences on sebum production in domestic animals described so far. For detailed literature please refer to the main text for the respective species

Cattle	▪ Influence of ambient temperature and humidity on sebum quantity and composition ▪ Increased sebaceous gland volume in Indian cattle in winter ▪ Slight reduction in sebum production in cows during the peripartum period ▪ Breed-dependent differences in sebum composition and sebaceous gland density ▪ Changes in sebum composition due to feeding and metabolic situation
Sheep	▪ Reduction of the glandular area with increasing age ▪ Increase in sebum production after shearing
Goat	▪ Influences of sex hormones during adolescence ▪ Seasonality of sebaceous gland size and activity especially in male animals
Pig	▪ Higher sebum production in animals with thicker back fat ▪ Breed differences
Horse	▪ Seasonal fluctuations in gland volume ▪ High sebum content on the skin of horses of the Curly Horse breed
Cat	▪ Decrease in the amount of sebum due to castration in male animals
Dog	▪ Increase in sebum production through supplementation of Saccharomyces cerevisiae fermentation product ▪ Atrophy and hypertrophy of sebaceous glands due to hormone-producing testicular tumors possible ▪ Breed-related differences in the amount of sebum on the skin and the proliferation of sebocytes

##### Nutritional influences

Energy deficiency leads to SG atrophy and reduction of sebum production with altered composition in humans (Pochi et al. 1970). In contrast, dairy cows, which usually suffer from an energy deficit in the peripartum, show only a slight decrease in size and cell proliferation of SGs during this period (Suzuki et al. 2018).

Higher proportions of C12:0 and C14:0 were detected in lipids obtained from hair samples of Holstein Friesian dairy cows with efficient energy utilization compared to animals with poor energy utilization, while the moiety of C16:0 was lower in animals with efficient energy utilization (Möller et al. 2019). This suggests a correlation between the composition of surface lipids and the metabolic situation. Another study also showed significantly higher levels of C12:0 in the hair lipids of Holstein Friesian cows with both, high reproductive performance and high milk yield (Moeller et al. 2013). Based on these observations the C12:0 content of cow hair might be a suitable biomarker for these performance parameters. Notably, both studies did not discuss the origin of the analyzed skin lipids.

Feeding Simmental cows high-energy diets in the second to sixth week of lactation led to higher levels of C18:1 and the n-3 FA α-C18:1 in the hair lipids between weeks four and eight of lactation (Wulf et al. 2022). The authors assumed that under higher energy levels, some of the FAs may have left the rumen unfermented and could, therefore, be incorporated directly into surface lipids.

##### Environmental influences

A seasonal change in SG volume was described in Indian cattle. The SGs in the inguinal region of the Deoni, Red Kandhari, Dangi and Gaolao breeds showed an increased volume and a higher density in winter (Rohankar et al. 2018). A number of studies reported an influence of temperature on sebum production and its compositions in male castrated Ayrshire calves (Smith 1975), Ayrshire bulls (Poon et al. 1978), hybrids of the British Shorthorn and Hereford breeds (*Bos taurus*) and American Brahman cattle belonging to the Zebus (*Bos indicus*) (O'Kelly and Reich 1982). Structural differences in the skin of Criollo Limonero, a cattle breed adapted to the warm Venezuelan climate, were also reported. Compared with zebus, which are also adapted to warm environments, samples from Venezuelan cattle showed significantly fewer HFs, sweat glands and SGs (Landaeta-Hernández et al. 2011). More recent studies compared the skin glands of heat-resistant Vechur cattle (a rare Indian breed of *Bos indicus*) with those of an undefined crossbreed (*Bos taurus*) (Naik et al. 2024), or Angus cattle and zebus of the Brahman breed (Mateescu et al. 2023). Both studies found a significantly higher number of SGs in the skin of the head, neck, abdomen and interdigital region in zebus compared to domestic cattle, which may be interpreted as a protective effect of sebum against heat stress and water loss through the skin.

Ambient temperature seems to be an important factor for the control of sebum production not only in adult cattle, as demonstrated by differences in the SG size of calves after exposure to heat in utero. Thus, exposition of pregnant cows to heat stress during the last 56 days of gestation resulted in a higher number of SGs in the calves at the time of birth, but the glands were of smaller size than those of the control group (Davidson et al. 2022). At 63 days after birth, calves in both groups showed approximately equal numbers of SGs, but the SGs of the calves exposed to heat in utero still were of smaller size than those of the control group. The authors suspected a temperature-dependent endocrine programming in the calves’ SGs during late gestation depending on the temperature environment.

#### Pathologies

##### Congenital diseases

Congenital diseases may affect the SGs in all species and usually appear directly at birth or shortly afterwards, and can be generalized or focal. They include congenital hypotrichosis (Scott 2018; Starič et al. 2019), dermoid sinus (Miller et al. 2012; Baumgärtner and Gruber 2020), panadnexal papillomatous hamartomas (Veiga et al. 2017), and ectodermal dysplasia (Kowalczyk-Quintas et al. 2014).

##### Neoplastic diseases

Tumors of the SGs are rare in cattle and appear predominantly in old animals. The jaw has been described as a typical location for sebaceous carcinoma, while sebaceous adenomas occur more frequently on the eyelids (Matovelo et al. 2005; Scott 2018).

##### Toxicological disorders

Intoxication of cattle with *Solanum glaucophyllum*, a plant of the nightshade family rich in vitamin D3 derivatives, leads to epidermal atrophy, including involution of HFs, sweat glands and SGs, probably due to the presence of vitamin D3 receptors on these structures (Gimeno et al. 2000).

##### Parasitic diseases

Parasitic infections affecting the SG are particularly important in cattle (Baumgärtner and Gruber 2020). The sebum composition appears to have an influence on the intensity of ectoparasitic infestation. For instance, tsetse flies (*Glossina spp.*) flew more frequently to traps equipped with sebum than traps without it, and showed an increased tendency to return to these targets after contact (Warnes 1989, 1995; Packer and Warnes 1991). Similar results were obtained in vivo for water buffalos (Gikonyo et al. 2000). In contrast, an inverse correlation between the amount of sebum on the skin and the number of horn flies (*Haematobia irritans*) infesting the animals was shown in cattle (Steelman et al. 1997). The specific composition of the sebum of cattle might be responsible for the good efficacy of levamisole, an anthelmintic often used as a pour-on, in this species. The permeability of bovine skin to levamisole in vitro is 400 times higher than that of human skin, arguably because transport takes place exclusively transcellularly in humans, while in cattle it takes place mainly via the appendages, i.e., HFs, sweat glands and SGs (Pitman and Rostas 1982).

Mites of the genera *Demodex bovis*, *D. ghanensis* and *D. tauri* occur as commensals in healthy cattle. If the immune system is impaired, clinical demodicosis may develop, usually on sparsely haired areas such as the neck, head, shoulders and chest. This can be triggered by a poor nutritional status, strong sun exposure, neoplasia, or treatment with corticosteroids (Slingenbergh et al. 1980; Deplazes et al. 2021). The parasites primarily infest the HFs, but can also accumulate in the SGs (Slingenbergh et al. 1980; Baumgärtner and Gruber 2020). In African buffaloes, infections with *D. cafferi* had a prevalence of 28% (Dräger and Paine 1980), and, as in cattle, masses of mites were detected in HFs and SGs. Chorioptic mange caused by *Chorioptes bovis* or *C. texanus* mainly affects the tail root region. A study showed that this region is especially rich in SGs with high amounts of anti-androgen binding protein beta-like (ABPβ-like), which acts as a mite attractant (Fujii et al. 2024).

Infestation of cattle with nematodes of the genus *Stephanofilaria stilesi* is common in North America. The nematodes are transmitted via haematophagous horn flies as intermediate hosts and adult nematodes are found in the HFs and in the SG excretory ducts (Lui et al. 2023).

##### Other infectious diseases

In dermatophilosis, a chronic skin infection with the gram-positive bacterium *Dermatophilus congolensis*, the affected SGs show necrotic sebocytes with loss of nuclei and cell borders (Amakiri 1974). The SGs of the skin adjacent to the hooves are affected in bovine digital dermatitis, gram-negative bacteria of the genus *Treponema* playing an important role in this disease (Evans et al. 2009). Lastly, *F. necrophorum* infection increased the epidermal thickness and number of hair follicles and sebaceous glands (Yue et al. 2025).

SG hyperplasia is observed in cattle infected with the pathogen causing lumpy skin disease, a virus belonging to the genus *Capripoxvirus*, subfamily Chordopoxvirinae, family Poxviridae (Trinh et al. 2022). In addition, involvement of the SGs has also been demonstrated in experimental infection of cattle with rinderpest virus (Wohlsein et al. 1993).

### Sheep

#### Structure of the gland

Wool fibers are hairs, but the word ‘wool’ is usually employed when describing the fine curly hairs that constitute the fleece produced by sheep and other species such as goat or yak (Rogers 2006). Sheep (*Ovis gmelini*) SGs differ considerably in size and function depending on whether they are in the hairy skin or in the woolly skin.

The SGs in hairy regions are larger than those in woolly regions, but the total area occupied by SGs is similar, as the density of primary follicles (i.e., those associated with large SGs and an arrector pilli muscle) in woolly regions is significantly higher than in hairy regions (Lyne and Hollis 1968). The highest SG density is found on the dorsal neck, a woolly region (Maya et al. 2017). In non-woolly (hairy or hairless) areas, particularly large SGs are located on the lips, vulva, anus and perineum (Lyne and Hollis 1968) (see also Table 2). In the dorsal perineal region, the glands are highly branched and have large excretory ducts (Kozlowski and Calhoun 1969). Small SGs open into the follicles of the facial vibrissae, whereas the hairless region between the nasal opening and the upper lip has no SGs (Lyne and Hollis 1968). A study on Merino sheep also revealed the presence of multilobular SGs in the udder skin, some of which opened into the HF with several ducts (Ludewig 1997). The SG morphology also varies depending on the association with primary or secondary HFs. Those associated with primary follicles are bilobular to multilobular and larger than those associated with secondary follicles, which have a variable number of lobules. Not all secondary follicles have SGs (Lyne and Hollis 1968; Kozlowski and Calhoun 1969). SGs were particularly evident in the sheep tail skin, and it was proposed that tail amputation could hinder physiological functions of these glands, such as intraspecies communication (Hümmelchen et al. 2025).

With increasing age, the hair density and the total SG area in sheep decreases (Vulov 1974). In adult sheep, however, the lipid layer is significantly thicker than in lambs, resulting in a higher resistance of the adult animals to the moisture-prone diseases "lumpy wool" and "fleece rot" (Warren et al. 1983). The thicker lipid layer in adult animals despite a smaller SG area might be caused by a better and longer accumulation of lipids on the skin of adult sheep, or because the SGs of lambs, though more numerous, may still be partially inactive (Warren et al. 1983). In Hetian sheep, testosterone treatment caused a significant increase in both the size and number of SGs (Feng et al. 2025). For a comparison between species regarding modulation of sebum production refer to Table 3.

The Meibomian glands in sheep have one main duct and several small ducts, which open directly onto the palpebral conjunctival epithelium (Kozlowski and Calhoun 1969). There are also other species-specific SGs in sheep that are particularly used for communication with conspecifics: the infraorbital organ, the interdigital glands and the inguinal glands (see also Table 2). Interestingly, these three types of specialized SGs are found in sheep but not in goats (Bosted et al. 2021).

The infraorbital organ is a skin invagination on both sides of the medial corner of the eye, in which both sweat glands and large, multilobular SGs are located (Kozlowski and Calhoun 1969; Bosted et al. 2021). The interdigital glands are located in the interdigital sinus on the dorsal side of the distal limbs, proximal to the interclavicular cleft. Superficially, the skin of the sinus contains many SGs, while deeper layers contain convoluted tubular glands, which are mainly responsible for the production of pheromones (Sivachelvan et al. 1992; Karahan et al. 2007; Alexandre-Pires et al. 2014). It has been suggested that the SGs in the interdigital sinus are not part of the interdigital gland, but merely serve as a component of the pilosebaceous unit (Pourlis 2010). In sheep of the South Indian Vembur breed, the interdigital glands were larger in males compared to females, suggesting that this difference was due to the influence of androgens (Rajagopal et al. 2022). The latter authors identified 23 volatile components in the secretion of the interdigital gland, at which the proportion of tetradecanol, tetradecanoic acid and hexadecanol was particularly high in both sexes. Octane, 7-hexyltridecane, tetradecane, heptadecane and decanoic acid were only found in females, while butanoic acid, 2-methylpropanoic acid, 1-heptanol and octadecanoic acid were only present in males. The authors hypothesized that these differences in composition inform about the sexual status to the opposite sex. However, it remains unknown whether these components were produced by the SG or the apocrine glands of the interdigital gland. Finally, in female sheep, the inguinal gland is located laterally at the base of the udder in the inguinal sinus (Bosted et al. 2021).

By injecting C14 acetate isotopes and subsequently measuring the radioactivity on the skin surface, the time between the synthesis of sebum lipids and their release was estimated to be about six days in sheep (Downing et al. 1975). Experiments using radioactively labelled lipids revealed that no flow of sebum occurs along the hair or wool fibers suggesting that sebum is only distributed in the coat as the hair or wool grows. This would make a replacement of lost sebum in fur or wool by freshly secreted sebum just as unlikely as a distribution via sweat (Hay and Mills 1982). The production and composition of sebum on the skin appears to be modified by shearing of Merino sheep. In the first two days after shearing, a 24% increase in sebum production compared to pre-shearing levels occurred. After four days, sebum production had returned to the initial level (Darwish et al. 1999). Furthermore, a rapid oxidation of the sebum components, particularly of free sterols, FAs and polar lipids occurred on the skin after shearing (Darwish et al. 1999).

Dermal administration of the antiparasitic agent deltamethrin resulted in better protection against ticks of the species *Ixodes rubicundus* in Merino sheep than in Dorper breed hair sheep (Kok et al. 1996). The authors assumed a different sebum content on the skin of the breeds as the cause of the different absorption of the active ingredient.

#### Composition of the sebum

The sheep sebum consists of 46% steryl monoesters and 10% wax monoesters, 12% each of free sterols and steryl diesters. Type 1 wax diesters account for 5% and type 2a wax diesters for 4%. The remaining 11% are unidentifiable or polar components (Wheatley 1986) (Table 1). The sterol fraction in the sheep species consists of 50% cholesterol, 34% lanosterol, 11% dihydrolanosterol, 5% desmosterol and 2% agnosterol (Wheatley 1986). The FAs are mostly branched-chain and/or have an odd number of carbon atoms. This distinguishes them from most other FAs found in other mammal tissues.

The bacterium *C. acnes*, involved as an opportunistic pathogen in the development of acne vulgaris in humans (Dréno et al. 2018), has a lipase that breaks down TAGs from sebum into FFAs and glycerol, which are then presumably used as nutrients by the bacterium. A negative correlation between the glycerol content on the skin surface and the presence of *C. acnes* exist in humans, indicating a high degree of utilization of glycerol (Rebillo and Hawk 1978). However, in the sebum of sheep only a very low proportion of TAGs was found, and *C. acnes* could not be detected (Webster et al. 1981).

A tumor-inhibiting effect on cells of Ehrlich ascites carcinomas of mice was demonstrated for ovine sebum FAs in vitro, particularly for those with a low boiling point. This effect occurred only after saponification, the untreated sebum from sheep did not show this effect. It was suggested that the α-carboxyl groups of the FFAs made an important contribution to the cytotoxic effect (Nakamura et al. 1994). Another study demonstrated a growth-inhibiting effect of fatty alcohols obtained from saponified ovine sebum on cells of Ehrlich ascites carcinomas in mice in vitro. The strongest effect was exerted by the most hydrophilic fatty alcohols with the lowest boiling points (Miwa et al. 1997).

#### Pathologies

##### Congenital diseases

Ichthyosis fetalis, characterized by hyperkeratosis, alopecia, and SG atrophy was reported in a newborn male lamb. The causative mutation was not identified (Câmara et al. 2017).

##### Neoplastic diseases

Spontaneous skin tumors are very rarely reported in sheep, probably because the dense wool cover prevents their detection. A trichoblastoma in a six-year-old female Sarda breed sheep, associated with focal SG hyperplasia and dilated ducts, was a notable exception (Polinas et al. 2020).

##### Parasitic diseases

Typical commensals on the skin of sheep include the mites *D. aries*, commonly found in HFs and the associated SGs and *D. ovis,* which infest the Meibomian glands and the SGs on the prepuce and vulva (Bukva 1990; Baumgärtner and Gruber 2020; Bosted et al. 2021). Clinically manifest demodicosis only develops when the animal's immune system is weakened. Then it manifests as itchy skin nodules (Bosted et al. 2021). Localised seborrhoeic dermatitis with hyperhidrosis and SG hyperplasia was reported upon infestation with mites of the species *Psoroptes ovis* (Mozaffari and Derakhshanfar 2009). Hair lice, although not infesting the SGs, presumably feed on their secretions (Bosted et al. 2021). Lipases were detected in the digestive system of *Damalina ovis* hair lice, a species specialized on sheep (Sinclair et al. 1989).

##### Other infectious diseases

A form of dermatitis in sheep (and also several other animal species), also known as lumpy wool disease or mud fever, is caused by the gram-positive bacteria *Dermatophilus congolensis* and/or *Dermatophilus dermatonomus* (Roberts 1963; Patten et al. 1995). Experimentally, a severe dermatitis could be induced by the application of *D. dermatonomus* in Merino sheep. However, lesions were restricted to skin areas previously degreased with petroleum, suggesting that the sebum film had a protective effect against this infection (Roberts 1963). This is consistent with the observation that natural infections with *D. dermatonomus* in Merino sheep usually occur shortly after birth or in lambs between three and twelve months of age, when the fatty film of the skin is not as pronounced as in adult animals (Warren et al. 1983). Besides its protective role for the skin surface, an inhibitory effect of the sebum on the growth of *D. dermatonomus* was also demonstrated in Merino sheep (Roberts 1963). The susceptibility to “fleece red”, a skin disease caused by the bacterium *Pseudomonas aeruginosa* that occurs more frequently after several days of rainfall was also increased after removing the skin sebum layer with petroleum (James and Warren 1979). Hyperplasia and hypertrophy of the SGs were described in “bolo disease”, which mainly affects female Merino sheep kept outdoors (Colly et al. 1990). Bacteria of the genus *Corynebacterium* could be isolated from the lesions, with which the clinical picture could be replicated experimentally (van Tonder et al. 1990).

In the viral disease “labial dermatitis” (also known as pustular dermatitis or ecthyma contagiosum), caused by *Parapoxvirus ovis*, the antigen is detectable in epidermal keratinocytes as well as in HFs and sebocyte precursor cells. This could explain the persistence of infectivity in recovered animals (Garrido-Fariña et al. 2008; Karki et al. 2019).

##### Diseases of other origin

Alopecia and hypoplasia of the sweat glands and SGs was observed in a phenomenon called Zhao Shen (most probably a sulphur deficiency) that affected a large number of sheep and goats in the Haizi region of China in the years up to 1998. (Youde and Huaitao 2001; Youde 2002). Traumatic injuries have been reported to large, exposed SGs such as the interdigital glands on the dorsal side of the claw. Additionally, a blockage of the gland opening by their oily secretion may also occur (Karahan et al. 2007).

### Goat

#### Structure of the gland

Structurally, SGs of goats (*Capra aegagrus hircus*) resemble those of other mammals (Jenkinson et al. 1985). The SGs of the primary hair follicles are well-developed and usually consist of two lobes opening on one side of the primary hair follicle, while those of the secondary hair follicles are underdeveloped, being composed of only a few sebocytes (Sultan and Abdulraheem 2020; Yang et al. 2025). Usually, SGs are detectable on 70% of all secondary hairs (Sağlam et al. 1994). The largest glands are localized on the primary hairs of the lateral abdomen, while the smallest glands are found on the ventral thorax. The SG density is highest on the dorsal neck and lowest in the lumbar region (Maya et al. 2017). Large SGs are also located on the snout, the scrotum, the inside of the limbs and the ventral side of the pinna (Table 2). An inverse relationship between the SG size and the density of the hair has been established (Sultan and Abdulraheem 2020). The planum nasale is free of SGs (Sar and Calhoun 1966).

Specialized SGs with a wide excretory duct are found throughout the teat skin, except for the area directly around the ostium papillare. Some of these glands are associated with hair and usually surround the follicle, while others are free glands. These specialized periareolar SGs show a higher density in the central part of the teat, compared to the base and tip. In addition, the glands are localized deeper in the dermis towards the tip of the teat, thus reducing their distance from the teat canal (Parmar et al. 1988; Parmar and Shrivastava 1990).

Further specialized free SGs in goats are located caudal to the horns in a fold of skin or, in unhorned goat breeds, at the base of the ear (Table 2). They are called cornual glands or intercornual glands and are responsible, among other aspects, for the strong odor of male goats (the "buck odor") (Smith et al. 1984; van Lancker et al. 2005; Dalga et al. 2021). The glands are located in the stratum superficiale of the dermis. They are branched and contain multiple lipid-filled vacuoles. They open into associated HFs and show hypertrophy in male goats during the mating season (Smith et al. 1984; Dalga et al. 2021). The glands also exist in females, but they are significantly less active (Smith et al. 1984).

The buck odor in male goats is also derived from subcaudal glands, located under the base of the tail in goats of both sexes (van Lancker et al. 2005). In billy goats, specialized SGs have also been described in the intermandibular region, the so-called mental glands, and on the prepuce. These glands are presumably used for pheromone production, too (van Lancker et al. 2005). Additionally, SGs are found in the external auditory canal of the goat. They are more numerous in goats than in other mammalian species. Together with the ceruminous glands, which are modified sweat glands, they produce cerumen (Wang et al. 2022)., which protects the auditory canal from chemical and physical injury and probably also has antibacterial properties. The SGs are located more superficially in the dermis than the ceruminous glands. (Yasui et al. 2004).

A study of goat fetuses revealed the presence of primordial bud-shaped SGs of the sinus hairs in the face of the animals as early as the 102nd day of gestation. These emerged from the peripheral part of the associated HFs and the root sheath. From the 114th day of gestation, the buds were visible all over the body (Kumar et al. 2019).

#### Influences on sebum secretion

The SG size and activity are influenced by several factors (Table 3). Non-castrated billy goats develop larger glands than castrated ones or females (Jenkinson et al. 1967). In Turkish Angora goats, SGs showed the highest activity and expansion in summer, thus, indicating seasonal changes in the gland activity (Sağlam et al. 1994). While no seasonal changes were observed in females, uncastrated male Saanen goats showed an enlargement of all SGs and changes in the composition of the sebum, particularly in the cranial half of the body, during the mating season in winter (Jenkinson et al. 1967). Goats appear to possess a lower overall amount of sebum on their skin than sheep. Presumably for this reason, treatment of Angora goats with the dermally administered antiparasitic agent deltamethrin under field conditions did not show the same effectiveness against an infestation with *Ixodes rubicundus* as in sheep (Kok et al. 1996).

The cornual glands of castrated billy goats responded to systemic application of testosterone with an increase in activity and enhanced pheromone production, while HF-associated SGs on the rump of the same animals showed no response (Iwata et al. 2000; Wakabayashi et al. 2000). In contrast to testosterone, dihydrotestosterone stimulated pheromone production in both the cornual glands and in unspecialized SGs on the rump in castrated males (Iwata et al. 2001). Therefore, the presence of the enzyme 5-α-reductase in the cornual glands, but not in the HF-associated SGs was suggested. Removal of the cornual glands resulted in a weakening of the billy goat odor, which is completely absent in castrated billy goats (van Lancker et al. 2005). The substance 6-trans-nonenal, identified as the odorant of the cornual glands, was produced during the mating season, but precursors were detectable in the glands at all times (Smith et al. 1984). High expression of elongation of long-chain fatty acids family member 5 (ELOVL5) and stearoyl-CoA desaturase 1 (SCD1), which are important for lipid synthesis, was detected in the SG basal cells in uncastrated billy goats at the time of pheromone production. The expression of the same genes was weaker in the cornual glands of either castrated bucks or female goats, and SGs on the rump of uncastrated animals showed no expression at all (Kitago et al. 2007).

#### Composition of the sebum

The lipids from the skin surface of goats have not been analyzed quantitatively so far (Table 1). Early studies reported the presence of cholesterol, cholesteryl oleate (an ester of cholesterol and C18:1) and C16:0 in the surface lipids obtained from goat skin (Nicolaides et al. 1968). Some unidentifiable components were interpreted as WE (Nikkari 1974). The surface lipids of Japanese goats and Saanen goats studied in the breeding season, contained free sterols and sterol esters (Sugiyama et al. 1986). FFAs were only present in significant quantities in adult billy goats. Here, the FAs in the sebum were mainly present as 4-ethyl FAs, whereas female animals showed a higher proportion of unbranched FAs. The extracted lipids made up 1–6% of the hair mass of the sampled region in billy goats and only 0.5–2% in females. In juvenile animals of both sexes, the proportion of lipids in the hair mass was about 1%. Glycoconjugates, presumably glycolipids, were detected in the SGs of Angora goats. N-acetyl-D-galactosamine, α-fucose, N-acetylneuraminic acid and N-glycolylneuraminic acid were found as saccharide residues. Neutral fats and PL were also present. In combination with the glycoconjugates secreted by the sweat glands, this establishes an alkaline environment in the fur of Angora goats (Meyer et al. 1993). The secretion of the SGs localized in the external auditory canal of Japanese miniature goats was shown to contain moderate amounts of glycoconjugates with saccharide residues of α-L-fucose, β-D-galactose, β-N-acetyl-D-glucosamine and N-acetyl-neuraminic acid (Yasui et al. 2004).

#### Pathologies

Diseases of the SGs in goats are uncommon and poorly documented. The most relevant seem to be infestations with parasites, although this rarely leads to clinical manifestations. In healthy goats, mites of the species *D. caprae* live as commensals in HFs and SGs; clinical symptoms mainly occur in immunocompromised animals. In this case, demodicosis manifests in the form of small skin nodules with crust formation and itching. Typical localizations are the eyelids, the prepuce, and the vulva (Zhao et al. 2013). Non-domesticated chamois are also affected by demodicosis, with an average prevalence of 2.6% in animals of the Italian Alps (Salvadori et al. 2016).

SG tumours are very rare in goats. Sebaceous adenomas usually affect adult animals, present as solitary protuberances, and can occur in any region of the body (Scott 2018). Only one report described a sebaceous carcinoma, which was located at the base of the horn, and two cases of sebaceous epitheliomas, both at the perianal region (Löhr 2013).

In goats, similar to sheep, infection with the *Parapoxvirus ovis* virus causes labial rash, also known as pustular dermatitis or ecthyma contagiousum. Infection with this highly contagious virus causes papules and pustules on mucocutaneous junctions (Garrido-Fariña et al. 2008). The viral antigen is detectable not only in epidermal keratinocytes but also in HFs and SG precursor cells. As in sheep, this could be a component of the viral persistence in animals which have been already recovered (Karki et al. 2019).

A disease that affected the SG in a large number of sheep and goats in the Haizi region of China until 1998, probably caused by sulphur deficiency, has already been discussed in the "Sheep" section (vide supra) (Youde and Huaitao 2001; Youde 2002).

### Pig

#### Structure of the gland

The SGs of pigs (*Sus scrofa domesticus*) are frequently described as rudimentary. They are smaller and surrounded by fewer blood vessels than in most other species (Mawafy and Cassens 1975; Meyer et al. 1978). Fittingly, the pig genome has partially lost genes responsible for sebum production (Lopes-Marques et al. 2019). Despite their small size, the SGs of pigs have a general structure comparable to other species. They consist of an unbranched alveolus and they are larger when associated with a growing HF (Mawafy and Cassens 1975). However, other authors described several lobules that form a narrow gland (Meyer and Görgen 1986). Pig Meibomian glands produce beta-defensins, which are important for the skin’s innate immune response (Marcarian and Calhoun 1966; Yasui et al. 2006). As in other mammalian species, free SGs are localized in the external auditory canal, which together with the apocrine ceruminous glands produce cerumen. Large SGs are located at the base of the inner side of the pinna occupying almost the entire dermis (Table 2). Their function is not known, but some authors assume that they prevent the penetration of water and bacteria into the auditory canal through large amounts of sebum (Meyer et al. 2001).

Pig skin is a popular model for human skin in biomedical and dermatological research (Lee et al. 2024). This is due to numerous similarities, such as sparse hair, a thick epidermis, well-developed rete ridges and papillary bodies, the presence of a thick subcutaneous fat layer and the absence of the panniculus carnosus (Schneider and Wolf 2016). However, porcine skin also differs from human skin in several aspects, including less pronounced vascularization, a thicker stratum corneum and clear differences in the structure and function of the apocrine glands (Meyer 1996). The skin of Yucatan minipigs was shown to display a wettability comparable to human abdominal skin, probably due to its low sebum content (Fujii et al. 2019). In other studies, the reactivity of the SGs with selected tissue antigens (Wollina et al. 1992) and the spatial distribution of enzymes were also comparable in the skin of humans and pigs (Neurand and Meyer 1976). The distribution of the enzymes varied according to the localization within the SGs. A moderate to strong activity of the numerous oxidative enzymes (cytochrome oxidase, malate dehydrogenase and glucose-6-phosphate dehydrogenase) could be observed in the SG peripheral cells, while enzymatic activity was weak to moderate in the centrally located maturing and mature sebocytes (Neurand and Meyer 1976). Hydrolytic enzymes such as β-glucuronidase, α-glucosidase, acidic and alkaline phosphatase as well as non-specific lipases and esterases showed a higher activity in mature and maturing sebocytes in the center of the SG than in the peripheral sebocytes (Moretti et al. 1966; Neurand and Meyer 1976). In the epithelium of the excretory duct and in the final sebum, activities of non-specific esterases and lipases were detectable. Taken together, the maturation of sebocytes might be divided into three phases based on the distribution of enzyme activities: In peripheral cells, the synthesis and processing of macromolecules requires high energy consumption and is supported the high activity of oxidative and hydrolytic enzymes. In the further course of differentiation, metabolism is reduced to a minimum and existing cell structures are broken down, whereby lysosomal enzymes such as acid phosphatase are particularly important. With the transit of sebum into the excretory duct, FAs are enzymatically released, as indicated by the pronounced activity of esterases and lipases (Neurand and Meyer 1976). The authors concluded that the SGs of pigs, despite being smaller, less numerous, and having a generally lower enzymatic activity, are in general comparable to those of humans in terms of their enzymatic equipment (Neurand and Meyer 1976).

The anlage of the SGs is first detectable in pig fetuses on the 60th day of development. By the 65th day, one to two layers of isoprismatic peripheral cells have already developed through proliferation and hypertrophy, while large, round, clearly granulated cells are present centrally. After 73–75 days of foetal development, the proximal part of the SG protrudes from the HF and the distal part forms a lumen in the HF, which later differentiates into the hair canal. The first SG lobules are formed around the 85th day of development (Meyer and Görgen 1986).

#### Influences on sebum secretion

A positive correlation between the amount of sebum present on the skin and the back fat thickness of pigs was reported (Luo et al. 2020) (Table 3), suggesting that the amount of sebum could be a suitable parameter for assessing body fat accumulation. Nevertheless it should be kept in mind that the relationship between back fat thickness and sebum quantity appears to contain a breed-specific component (Luo et al. 2020).

#### Composition of the sebum

In contrast to the other mammals studied so far, the majority of the lipids that cover the skin surface of pigs do not originate from the SG but are of epidermal origin (Nicolaides et al. 1968). The lipid content on the pig skin was shown to represent only one tenth of that of densely haired mammals (Nicolaides et al. 1968). Also in contrast to other mammalian species examined, the lipids obtained from these areas did not contain any WE or diesters; but included sterol esters, free sterols, FFAs and TAGs (Nicolaides et al. 1968; Lindholm et al. 1981) (Table 1). In tissue sections of pig skin, the lipids within the sebocytes contained very small amounts of neutral and acidic glycoproteins and glycogen (Tsukise and Meyer 1983). Additionally, WEs and TAGs, containing unsaturated FAs, were detected in sebocyte intracellular lipid droplets (Zvára and Hradil 1974). Glycogen could not be detected in the sebum, but was present in the basal cells as indicated by positive PAS reaction (Zvára and Hradil 1974).

Degradation products of pharmacologically active substances can occur as further components in the porcine sebum. For example, the sodium salt of diclofenac (a non-steroidal anti-inflammatory drug) remains detectable in the sebum more than 14 days after intramuscular injection, a time point when it had already been eliminated from all other tissues. Therefore, the withdrawal period from administration of the drug to the production of food from these animals was set at 15 days (Li et al. 2019). The synthetic progestin analogue, Altrenogest, which is administered orally for estrus synchronization in sows, also passes into the sebum, although significantly higher concentrations are detectable in the liver and kidneys (Li et al. 2021).

#### Pathologies

Focal cutaneous hypoplasia, a congenital ectodermal defect, is the only well-documented disease involving the SG in pigs. It occurs very rarely, affecting the Essex and Large White breeds and crossbreeds. The disease is characterized by a hypoplastic epidermis and loss of HFs and SGs, resulting in alopecia and depigmentation in the affected areas (Scott 2018). Apart from this congenital disease, no other SG pathology in pigs has been described, which is consistent with the concept that pig SGs are rudimentary.

Classical swine fever is an important infectious disease that does not cause changes in the SG but is spread via their secretions. The virus was detectable 28 days post infection in HFs, sweat glands, and SGs, including the epithelium of the excretory ducts, of infected pigs (Oki et al. 2022). The authors concluded that the virus may spread via the skin surface, and considered skin and hair as possible sample material for diagnosing the disease (Oki et al. 2022).

## Sebaceous gland and sebum function in animals domesticated for work

### Horse

#### Structure of the gland

In horses (*Equus caballus*), the SGs consist of two to eight lobules. The largest and most numerous SGs are found at mucocutaneous junctions, including the eyelids and the teats. Prominent accumulations of SGs are also present on the mane ridge, in the submandibular region and on the coronet band (Scott and Miller 2011) (Table 2). Maturing sebocytes in horses were reported to contain multiple mitochondria as well as hexagonally arranged crystalline lattices, presumably representing catalases associated with peroxisomes (Jenkinson et al. 1985). Precursor cells and basal cells together account for about 10% of the gland volume in equine SGs, while the maturing sebocytes and the degenerating sebocytes make up about 55% and 35% of the gland volume, respectively (Jenkinson et al. 1985).

During the foetal development of horses, the SG precursors are first visible on the eyelids approximately on gestation day 150, which corresponds to a crown-rump length (CRL) of 27 cm (Fechter 1914; Kressin and Brehm 2019). Around day 175 of gestation (CRL of 35 cm), first SG precursors were also detected in the tail skin and around day 180 of gestation (CRL of 38 cm) the formation of sebaceous ducts was reported in the skin of the lips (Fechter 1914; Kressin and Brehm 2019).

#### Influences on sebum secretion

The volume of the equine SGs undergoes seasonal adaptations. In thoroughbred horses, an increase in the volume of the glands was observed in winter, while in non-thoroughbreds the volume density (the mass of glands per unit volume of the subcutis) was higher in summer. Ponies showed no seasonal differences in the volume and density of their SGs. Such seasonal differences might be related to a water-repellent protective action of sebum in winter and a wicking effect of sebum-covered hair on sweat in summer in thoroughbreds and non-thoroughbreds respectively (Sneddon et al. 2008). High sebum content was found in Curly horses, possibly leading to an increased binding of allergens from dander and sweat (Zahradnik et al. 2018). Table 3 provides a comparison between the different species regarding modulation of sebum secretion.

#### Composition of the sebum

The composition of the equine sebum is unique compared to other domestic mammals (Table 1). It contains large amounts of large-ring lactones (48%), stearoyl monoesters (38%) and free sterols (14%) (Wheatley 1986). The relative amounts of the components vary only slightly between different equine species (Lindholm et al. 1981).

The most unique components of the equine sebum are lactones from methyl-branched omega-hydroxy acids, consisting of 32, 34 or 36 carbon atoms (Downing and Colton 1980; Lindholm et al. 1981). These large-ring lactones can be hydrolyzed into omega-hydroxy acids with chain lengths of mainly 33, 35 or 37 carbon atoms. Most (78%) of these omega-hydroxy acids are monounsaturated, 16% are saturated and 6% contain two double bonds. The double bonds of the different monounsaturated omega-hydroxy acids are located in roughly equal proportions at either positions n8 or n10. In contrast to the lactones derived from horses, sebum lactones of donkeys consist mainly of unbranched chains, while the sebum of mules (hybrids between a donkey and a horse) contains about 50% each of lactones from branched and unbranched chains. The function of large-ring lactones, which have not yet been detected in the sebum of any other species, has not yet been definitively clarified. Some authors suggested a pheromone effect, despite the large size of the molecules and their low volatility (Downing and Colton 1980; Wertz et al. 1983; Wertz 2018).

Lipids in the horse sebum are likely to be synthesized de novo in the sebocytes, as sebum lipids are mainly branched-chained while lipids circulating in the blood of horses consist almost exclusively of unbranched FAs (Colton and Downing 1985). About five weeks elapse between the synthesis and secretion of the lipids. The synthesis presumably takes place in two steps, which include the intermediate storage as PL. This assumption was based on the observation that phosphatidylcholine was detectable in large quantities in mature sebocytes, but was completely absent in the final secretion (Colton and Downing 1985).

Beside the large ring lactones, stearoyl monoester make up a large proportion of equine sebum lipids. Roughly half of the stearoyl monoesters have chain lengths of 22 or more carbon atoms, and the majority (approx. 90%) are branched chains. Monounsaturated molecules account for 46.7% of the stearoyl monoesters (Wheatley 1986).

The species-specific composition of the sebum of horses appears to be important in interaction with ectoparasites. It was shown that flies of the species *Gasterophilus intestinalis* laid their eggs on human arms if these were previously rubbed against the flanks of horses, which probably covered the skin with equine sebum (Cogley and Cogley 2000). In contrast to this attractive effect on flies, a repellent effect on the tick *Amblylomma sculptum* was demonstrated for the sebum of donkeys but not for equine sebum. The odorant (E)−2-octenal which is found in the sebum of donkeys but not in the sebum of horses seems to be responsible for this repelling effect (Ferreira et al. 2019).

#### Pathologies

Pathologies of equine SGs are rare. A few case studies exist, describing sebaceous adenitis (Osborne 2006; Lorch et al. 2013) or calcification and osseous metaplasia of the Meibomian gland (Gunsalus et al. 2023). Tumors of the SGs are also rare and usually affect adult to old horses. In most cases they are benign and grow slowly, but show a tendency to ulcerate early (Knottenbelt 2009). However, sebaceous carcinomas showing invasive growth and metastasis have also been described (McMartin and Gruhn 1977; Aydın Gürel et al. 2018).

Besides sebaceous adenitis and tumors, primary and secondary seborrhea occurs in horses (Knottenbelt 2009; Scott and Miller 2011). Additionally, changes in the SGs can be observed in numerous other equine dermatoses. These include atrophy or cystic enlargement of the glands in inflammatory processes in the dermis or developmental dermatoses. SG hyperplasia is also possible in the context of chronic inflammatory skin diseases. SG melanosis can be observed in follicular dysplasia, alopecia areata and demodicosis (Scott and Miller 2011). The mites of *D. equi* can be found not only within HFs but also in SGs (Deplazes et al. 2021).

## Sebaceous gland and sebum function in animals domesticated for companionship

### Cat

#### Structure of the gland

In cats (*Felis catus*), SGs are rather small, consist of two to three lobules on the back and lateral chest wall, and are unbranched on the abdomen and forechest (Wienker 1968; Affolter and Moore 1994; Charpin et al. 1994). For cats (and dogs), it was proposed that the secretion of sebum through the excretory duct into the follicular isthmus is supported by contraction of the arrector pili muscle (Kristensen 1975). However, experimental evidence is lacking. The highest SG density is found at mucocutaneous junctions, between the toes, on the dorsal neck and trunk, on the dorsal root of the tail and on the chin, while the planum nasale and the footpads have no SGs (Wienker 1968; Affolter and Moore 1994) (Table 2). The blood supply to the SGs is more pronounced in cats than in humans or pigs (Meyer and Neurand 1977).

The anlage of the cat SGs are formed during embryonic development by sprouting from the follicular epithelium (Affolter and Moore 1994). This occurs during the keratinization process of the hair through protrusions of large, translucent cells on the sides of the bulb cone in its upper third in fetuses of approx. 10.5 cm in length (Backmund 1904), which corresponds to approximately the 20th day of embryonic development.

Cats have various specialized SGs (Table 2). The large SGs in the chin region are known as the mental organ, which cannot be clearly distinguished from the circumoral organ localized around the cleft of the mouth (Kristensen 1975; Salomon et al. 2020). The circumoral organ also mainly consists of SGs, but also contains tubular glands (Salomon et al. 2020; König and Liebich 2024). Cats have large, free SGs between the toes (glandulae tori), but their function is unknown (König and Liebich 2024). Free SGs are also found in the external auditory canal. They are located in the connective tissue of the dermis, are surrounded by a thin basement membrane, and their excretory duct opens directly onto the skin surface (Amemiya and Okazoe 1960). Interestingly, peripheral cells of SGs in the external auditory canal of cats contain glycogen (Fernando 1965). Other specialized skin glands of cats, as the supracaudal gland (Affolter and Moore 1994) and the anal sac glands (Sokolov and Shabadash 1979; Shabadash and Zelikina 2003), have been considered by some authors as SGs but are in fact hepatoid glands (Affolter and Moore 1994), which, in contrast to SGs, form their ducts de novo by lysis of glandular cells (Shabadash and Zelikina 2003).

#### Composition of the sebum

In domestic cats, 66% of the sebum consisted of type 1 wax diesters, 3% of free sterols and 31% of unidentifiable or polar components (Wheatley 1986) (Table 1). Other authors (Thody and Shuster 1989) also found a small proportion of wax monoesters in the sebum of cats. The composition of sebum varies strongly within the Felidae family. Analysis of the surface lipids on the skin of bobcats (*Lynx rufus*) and European wildcats (*Felis sylvestris*) using thin-layer chromatography revealed clear differences in the composition of the lipids, but the components were not categorized more precisely (Lindholm et al. 1981).

A number of studies assessed the activity of non-specific esterases, lipases, and acidic and alkaline phosphatase in cat SGs (Meyer and Neurand 1976; Neurand and Meyer 1976; Wheatley 1986). A special feature in the enzyme equipment of cat SGs was the detection of a weak arylsulfatase activity in the sebocytes and a moderate to strong activity of this enzyme in the final sebum. Arylsulfatases are usually found in lysosomes, where they are involved in the degradation of sulfatides (hydrogen sulfate esters of glycosphingolipids, which are mainly found in cells of the central nervous system). The detection of the enzyme might be a possible indicator for the presence of sulfatides in the sebum of cats, even if these could not be detected (Meyer and Neurand 1976). In contrast to human SGs, no clear activity of the enzyme β-glucuronidase was detectable in the SGs of cats. The authors surmised that this enzyme degrades androgens, which might correlate its activity to the sexual cycle (Meyer and Neurand 1976). Regarding oxidative enzymes in the cat SGs, there was a very strong activity of glucose-6-phosphate dehydrogenase in the peripheral cell layers as well as moderate to strong activities of the enzymes isocitrate dehydrogenase, lactate dehydrogenase and NAD(P)H diaphorase (Meyer and Neurand 1977). In the central cell layers, strong activity was only detectable for glucose-6-phosphate dehydrogenase, while the other oxidative enzymes showed only low to moderate activity (Meyer and Neurand 1977). In another study, PL were detected in the peripheral sebocytes of the external auditory canal in addition to neutral lipids (Fernando 1965).

Another important component of cat sebum is the protein complex Fel d 1 (Felis domesticus allergen 1, formerly cat allergen (CA)1), which is the trigger for the most common allergy to pets in humans. It is excreted by cats mainly via sebum and saliva, but small amounts of the allergen are also produced by basal epithelial cells of the epidermis (Charpin et al. 1991; Shah and Grammer 2012). The protein is not detectable in the blood serum and urine. Brushing not only applies saliva to the coat, it also favors the distribution of sebum and thus the allergen in the animal's coat (Charpin et al. 1991). The initial hypothesis that the origin of Fel d 1 in the fur of cats originated exclusively from the saliva was refuted by demonstrating high concentrations of the allergen in SGs and at hair roots (Bartholomé et al. 1985). Other authors found a strong correlation between the content of Fel d 1 in the fur and the SG density in the corresponding skin area (Mata et al. 1992). In addition to sebum and saliva, Fel d 1 is also found in tear fluid and the anal glands. In the latter an even higher concentration of Fel d 1 was detected compared to sebum (Andrade et al. 1996; Thoms et al. 2019). In tomcats, the amount of Fel d 1 in the fur could be reduced by more than two thirds by castration. However, the initial state could be temporarily restored by injecting testosterone (Zielonka et al. 1994), which is in line with the reduction of the amount of sebum produced after castration (Charpin et al. 1994; Zielonka et al. 1994) (Table 3). However, it is not clear whether the production of Fel d 1 is directly subjected to hormonal control or whether the reduction in the Fel d 1 content was caused by the overall reduction in sebum production. A new approach for the prophylaxis of cat allergy in humans in the form of vaccination of cats against the Fel d 1 protein was developed by injecting cats with serum containing recombinant Fel d 1 bound to an immunologically optimized carrier molecule, resulting in long-lasting and strong antibody production (Thoms et al. 2019). Along this line, dietary interventions by feeding cats with avian immunoglobulins isolated from egg yolks might also reduce Fel d 1 levels (Colosimo et al. 2025).

The sebum of cats may also play a role in the distribution of pharmaceuticals on the skin. The antiparasitic agent tetrachlorvinphos, which is contained in collars against flea and tick infestation, showed high solubility in the sebum of dogs and cats, suggesting that it is distributed over the body surface by the sebum flow (Driver et al. 2024).

#### Pathologies

##### Inflammatory diseases

Sebaceous adenitis, the inflammation of the SG, usually affects cats in middle adulthood. It is typically localized at the head, neck and trunk (Wendlberger 1999; Inukai and Isomura 2007; Possebom et al. 2015; Glos et al. 2016), and involves alopecia as well as crusts and scales (Wendlberger 1999; Sepibus et al. 2004; Noli and Toma 2006). It can be idiopathic or secondary to other diseases. As most cases of cat sebaceous adenitis have no triggering primary disease, an autoimmune cause is suspected (Wendlberger 1999). Exfoliative dermatitis, a paraneoplastic skin disease associated with thymoma, is characterized by inflammatory infiltration of SGs and possibly replacement by inflammatory cells, occurs rarely (Rottenberg et al. 2004; Linek et al. 2015). Inflammation also affects the cat Meibomian gland as chalazion, a nodular elevation visible on the conjunctiva, which can be the cause of conjunctivitis due to mechanical irritation. Otitis externa is another inflammatory disease involving the SG. It usually affects both, the vertical and horizontal parts of the external auditory canal and is accompanied by SG hyperplasia and dilatation of the excretory ducts in the acute stage (van der Gaag 1986).

##### Congenital diseases

Congenital disorders of the SG are very rare in cats and can be generalized, such as SG dysplasia and seborrhoea, or localized, such as hamartoma, sebaceous nevus and dermoid sinus. SG dysplasia is characterized by progressive hypotrichosis up to alopecia, and has been described almost exclusively in shorthair cats. The first symptoms usually appear between the 4th and 12th week of life and include dandruff and crust formation in varying degrees as well as thin fur (Yager et al. 2012; Peters-Kennedy et al. 2014; Kiener et al. 2023). The SGs are reduced in size and irregular in shape. No clear differentiation of the sebocytes, but rather an accumulation of undifferentiated basal cells, occasionally with lymphocytic inflammation can be observed. Neither pruritus nor changes in blood chemistry occur, making an inflammatory cause or endocrine disorders unlikely (Canadas et al. 2018). A genetic cause, in particular mutations in the gene encoding the enzyme Sterol O-acyltransferase 1 (SOAT1) is suspected, but has so far only been proven for individual cases (Kiener et al. 2023). A rare congenital form of greasy seborrhoea of unknown origin has been described in Persian and Himalayan cats (Noli and Scarampella 2004).

The SG hamartoma appears in the form of non-inflammatory solitary, hairless skin nodules and is usually localized on the limbs or trunk. The nodules consist of large, randomly arranged lobules with normal sebocytes (Gross et al. 2005). In SG nevus, glands are moderately enlarged and very numerous in one area of the dermis. The skin surface has an irregular appearance and is greasy, and HFs are underdeveloped, leading local alopecia (Gross et al. 2005). Dermoid cysts, in contrast, are characterized by a hollow mass lined by SGs (Tong and Simpson 2009; Fleming et al. 2011; Akhtardanesh et al. 2012; Koch et al. 2022).

##### Neoplastic diseases

Different types of SG tumors have been reported in cats, including sebaceous adenomas (Levene 1984; Ramos-Vara et al. 2002) and sebaceous carcinomas (Martín de las Mulas et al. 1995; Sozmen et al. 2002; Kapakin et al. 2008). Their histological features are described in more detail in the section concerning dogs. Such SG tumors are rarely observed in cats and usually occur in older animals, with no gender or breed predispositions (Scott and Anderson 1992; Gross et al. 2005).

##### Diseases of nutritional or toxicological origin

Two cats developed skin lesions consisting of bilateral symmetrical hair loss and an almost complete absence of SGs on the abdomen and limbs two weeks after accidental exposure to diesel fuel (Declercq and Bosschere 2009). A lack of essential FAs in the diet can lead to long-term dermatological symptoms in domestic cats with a generalized reduction in sebum, which leads to a dull coat, dry skin with dandruff, and alopecia (Muller et al. 1993). As the deficiency progresses, sebum production increases significantly, affecting the outer ear canals and the areas between the toes in particular. The skin and coat appear greasy, the skin is thickened and bacterial pyoderma may occur (Muller et al. 1993).

##### Parasitic diseases

In the case of severe infestation, Demodex mites can also occur in SGs, although this happens almost exclusively in immunocompromised animals (Baumgärtner and Gruber 2020). In rare cases, an infestation with mites of the species *D. canis* can also be detected (Sastre et al. 2016). *D. cati* usually infects the eyelids, temples and chin of its host animal; wide infestation is possible in cases of severe immunosuppression. Infections with *D. gatoi* are usually localized on the neck, shoulders and thorax (Peters 2016). A case of otitis externa caused by *D. cati* was reported in a cat tested positive for the feline immunodeficiency virus (Milley 2018).

##### Viral diseases

Multiple large crusty plaques in the left preauricular region, on the bridge of the nose and in the right periocular region associated with clearly hyperplastic SGs were reported in a 15-year-old shorthair tomcat (Munday et al. 2017b). DNA from papilloma viruses was isolated from all infected areas, and a DNA sequence identical to the FdPV-MY sequence was amplified, which was suspected to originate from a presumably unclassified papilloma virus type. The authors classified the papilloma virus as Felis catus papillomavirus 5 (FcaPV-5) (Munday et al. 2017a).

##### Diseases of unknown origin

Acne-like lesions, including comedones, which can progress to papules and pustules, as well as localized alopecia often occur in the chin region of cats. The disease is known as feline acne. Unlike acne in humans, there is no link to adolescence. Consequently hormonal causes seem not to be involved in the pathogenesis (Muller et al. 1993; Plewig and Kligman 2000). The disease occurs in cats of all ages without gender predisposition (White et al. 1997; Jazic et al. 2006). Feline acne may develop due to the poor accessibility of the chin region by the cat during grooming, leading to the accumulation of grease and dirt, making the skin susceptible to comedones (Muller et al. 1993). Another disease of the SGs in cats with an unknown etiology is sebaceous retention cysts, which may be caused by obstruction of a HF and, thus, also of the sebum drainage pathway (Muller et al. 1993).

### Dog

#### Structure of the gland

In dogs (*Canis lupus familiaris*), HF-associated SGs show the basic structural pattern observed in other mammals (Lovell and Getty 1957). As in cats (see above), secretion of sebum through the excretory duct into the follicular isthmus has been suggested to be supported by contraction of the arrector pili muscle (Kristensen 1975). In addition, the SGs of dogs and cats show a pronounced blood supply (Meyer et al. 1978). The highest density of canine SGs is found at mucocutaneous junctions, between the toes and on the dorsal neck and trunk (Table 2). The footpads and the nasal planum are free of SGs (Lovell and Getty 1957; Affolter and Moore 1994). Expression of senescence-associated β-galactosidase was reported in the SGs of old dogs, but detailed information regarding the age of the animals is not available (Pati et al. 2014). Aquaporin 3, but neither aquaporin 1 nor 5, was detected in the SG basal layer, suggesting a role for aquaporin 3 in SG homeostasis (Sonoda et al. 2024).

The canine SGs are formed during embryonic development by sprouting from the epithelium of the HF, whereby excretory ducts open into the follicular isthmus. In contrast, at the partially hairless skin of the lips, eyelids, external auditory canals and anal region, free SGs develop by sprouting from the epidermis with ducts opening directly onto the skin surface (Affolter and Moore 1994; Wang et al. 2022).

Specialized SGs in dogs include a rudimentary dorsal tail gland consisting of large HF-associated SGs, which is more pronounced in males (König and Liebich 2024) (Table 2). The circumanal glands of canids consist mainly of hepatoid glands with only a small number of apocrine glands and SGs opening into HFs. In females, the hepatoid glands are less pronounced, but the proportion of apocrine glands and SGs is larger than in males. However, whether SGs contribute to the function of the circumanal gland remains questionable (Shabadash and Zelikina 2002; Chernova 2012; Brodzki et al. 2014, 2021). Free SGs are found in the external auditory canal of dogs in addition to ceruminous glands (Wang et al. 2022). Compared to other species, these SGs are localized closer to the ceruminous glands and situated within the same skin layer, possibly enabling a synchronous release of secretions (Wang et al. 2022). In Anatonial shepherd dogs, large numbers of free SGs are located directly under the epithelium of the lamina propria in the rostral parts of the ventral and dorsal nasal conchae (Abumandour et al. 2022).

In African wild dogs, but not in domestic dogs, a high density of specialized SGs is localized in the skin of the cranial prepuce (van Heerden 1981). These glands, presumably responsible for the olfactory labelling of the urine, open partly into hair shafts and partly directly onto the skin in the lumen of the prepuce (van Heerden 1981).

#### Influences on sebum secretion

A recent study on 149 dogs grouped into four different age groups, four different sex groups and five different breedings, did not show any influence of these parameters regarding the sebum level (Kwon et al. 2025). Some studies showed an influence of feed additives on the amount and composition of sebum in dogs. Supplementing English Pointer dogs with the *Saccharomyces cerevisiae* fermentation product (SCFP) increased sebum production (Wilson et al. 2022). In contrast, supplementation of Beagles for eight weeks with ethyl esters of omega-3 FAs from linseed oil led to a decrease in the proportion of saturated FAs and short-chain FAs and an increase in the proportion of polyunsaturated FAs in sebum (Wyrostek et al. 2023). This was particularly due to increased levels of the omega-3 FA α-linolenic acid and its derivatives eicosapentaenoic acid (20:5) and docosahexaenoic acid (22:6). The changes in sebum composition persisted eight weeks after the end of supplementation. In another study, no differences in the amount of sebum or skin moisture were detected after feeding different diets (Geary et al. 2022). Hormone-producing tumors are known to influence sebum production in male dogs. Thus, Sertoli cell tumors can lead to generalized SG atrophy in the affected dogs, while testicular tumors (which produce androgens) can result in SG hyperplasia and greasy skin (Muller et al. 1993). Table 3 provides an overview onto the impact on sebum production in different species.

#### Breed-specific features

Mexican Hairless Dogs with completely hairy bodies show no differences regarding the histological skin structure compared to Beagles (Kimura and Doi 1994). In hairless individuals of this breed, fewer HFs and therefore fewer SGs are present, even on the head and tail which are occasionally covered with hair (Fukuta et al. 1991; Kimura and Doi 1994). In the non-haired skin of Mexican Hairless Dogs, the thickness of the epidermis, as well as the number of HFs and SGs decreases with increasing age (Fukuta et al. 1991). When comparing different breeds, the highest amount of sebum was found on the skin of Labrador Retrievers, followed by Manchester Terriers and Fox Terriers, while Beagles showed the lowest amount (Young et al. 2002). Likewise, the proliferation of sebocytes varies between breeds, as a significantly lower value was reported in Cocker Spaniels compared to Beagles, even if no differences in size or structure of the SGs were visible between the two breeds (Kwochka and Rademakers 1989). Border Terriers have a higher number of both basal cells and differentiated sebocytes in the individual lobules of their SGs than other terrier breeds (Dedola et al. 2010). In contrast to the observations of Young et al. 2002, no differences regarding the amount of sebum in the inguinal region was found neither between sexes nor between different breeds (Kwon et al. 2025). However, in the study of Kwon et al. (2025), other breeds (Beagle, Cocker spaniel, Maltese, Pomeranian, Miniature poodle) were examined as in the study of Young et al. (2020). Also, besides differences in the studied breeds, sebum was measured on a shaved patch in the lumbar region (Young et al. 2002) or in the unshaved inguinal region (Kwon et al. 2025), making a direct comparison of the studies problematic.

#### Composition of the sebum

The composition of sebaceous lipids is very similar within the Canidae family (Lindholm et al. 1981). Thin layer chromatography revealed that sebum of domestic dogs consists of 35% wax diesters type 2a. Most of them are long-chain 1,2-diols, each esterified with a long and a short FA. Further, there are 42% stearoyl monoesters, 9% free sterols and 12% of unidentifiable or polar components (Lindholm et al. 1981; Wheatley 1986) (Table 1). The stearoyl monoesters in the canine sebum consist of 83.2% branched-chain FAs, 9.8% unbranched FAs and 7% monounsaturated FAs. When considering all FAs, 19% have a chain length of 22 or more carbon atoms. The saturated FAs of the type 2 wax diesters are 20.7% unbranched and 79.3% branched. Of these FAs, 17.3% consist of at least 22 carbon atoms (Wheatley 1986). Canine sebum lacks significant amounts of TAGs. Although the proportions of sebum lipids varied between different species within the Canidae family, no differences between sexes or between different dog breeds were found (Lindholm et al. 1981; Burton et al. 1992).

Based on the expression of the gene *CBD103*, which codes for the protein β-defensin 103, an immunological function was suggested for canine sebum (van Damme et al. 2009). β-defensins are small, cationic peptides that play an important role in non-specific immune defense. They exert an antimicrobial effect through interaction with anionic membrane components, such as acidic PL (e.g., phosphatidylglycerol or -serine), of bacteria (Toke 2005).

Hairless crossbreeds of Mexican Hairless Dogs and Beagles show local acne like symptoms like comedones, pustules, milia and abscesses (Bedord and Young 1981). The lipids originating from the affected skin areas consisted mainly of free sterols. About 55% to 65% of these sterol esters had a chain length between 22 and 26 carbon atoms. In addition, α-hydroxypalmitic acid and the unsaturated FAs C16:1, C18:1 and C18:2 which are not found in healthy skin, were detectable in significant amounts. Surface lipids from healthy skin areas of the same dogs consisted mainly of WE and sterol esters; free sterols were also detectable in small amounts (Bedord and Young 1981).

Skin lipids of large poodles suffering from sebaceous adenitis showed lower levels of wax diesters and cholesteryl esters than skin lipids of healthy poodles (Burton et al. 1992). Higher amounts of WE and TAG, but relatively fewer FFA and epithelial ceramides were detected in the skin lipids of Shi Tzus with seborrhoea compared to healthy dogs of the same breed (Yoon et al. 2013). In West Highland White Terriers, skin lipids did not differ between dogs suffering from atopic dermatitis and healthy individuals, but varied between affected and unaffected skin areas of the same dogs (Orbell et al. 2022). However, the strong differences detected between the individuals may have masked possible differences between diseased and healthy dogs.

Canine Meibomian glands might play a role in maintaining the correct pH value of the tear film, as they express the isoenzymes carbonic anhydrase II and IV, which are necessary for the production of bicarbonate (Sugiura et al. 2010). However, the authors did not exclude the possibility that carbonic anhydrase IV could also play a role in lipogenesis.

As in other species, the sebum of dogs plays an important role in the external treatment with antiparasitics, as these agents accumulate in HFs and SGs. For instance, radioactively labelled imidacloprid and fipronil could be detected in HFs and SGs up to one month and up to 56 days respectively (Cochet et al. 1997; Chopade et al. 2010). Tetrachlorvinphos (see above) might be easily distributed over the body surface because of its high solubility in sebum (Driver et al. 2024). Another drug that spreads over the skin surface via the sebum is the antimycotic terbinafine, administered orally to dogs to combat malassezia-related dermatitis. However, the accumulation of this drug in both the sebum and the epidermis is extremely low compared to the serum level, requiring high oral doses to achieve an effective level in the skin (Gimmler et al. 2015).

Excretion via the sebum has been demonstrated in dogs for the chemotherapeutic agent carboplatin after systemic application, which might represent a potential health risk for veterinary staff, owners, and partner animals (Janssens et al. 2011, 2015). On the other hand, measurement of the active ingredient content in sebum can also be used as a tool to monitor systemic active levels and thus the therapy (Janssens et al. 2011).

#### Pathologies

##### Inflammatory diseases

Idiopathic sebaceous adenitis is one of the most frequent immune-mediated pathologies of the SGs in dogs (Muller et al. 1993). It mostly affects middle-aged dogs, with no clear gender predisposition, although some studies show a slight overrepresentation of male animals (Muller et al. 1993; Pye 2021). The Magyar Viszla, Akita, Great Poodle, Havanese, Springer Spaniel, Chow Chow and Samoyed breeds are frequently affected (Muller et al. 1993; Müller 2000; Spaterna et al. 2003; Sousa 2006; Lam et al. 2011; Baumgärtner and Gruber 2020; Shumaker 2020). In Akita and large poodles, an unidentified autosomal recessive hereditary defect is suspected to be the cause of the disease (Sousa 2006; Baumgärtner and Gruber 2020; Pye 2021). Macroscopically, the disease manifests as a focal to generalized alopecia with hyperkeratosis, scaling, erythema and keratin cuffs. Hairs are surrounded by a sheath of keratin-containing deposits that persists during hair growth (Sousa 2006; Pye 2021). Granulomatous or pyogranulomatous infiltrates of the SGs are present in the affected areas in early stages, they can completely replace the SGs as the disease progresses (Muller et al. 1993; Pye 2021). Due to the reduced or lacking sebum production, the antimicrobial protection of the skin is strongly reduced (Pye 2021). An inflammation of the SGs resembling idiopathic sebaceous adenitis also occurs in spontaneous, non-infectious alopecia of Norwegian Lundehund dogs. However, the pathogenesis of the two diseases differs. The inflammatory processes in the Lundehund are mainly directed against HFs, with only a secondary alteration of SGs (Bergvall and Shokrai 2014). Apart from skin alterations, a reduction of tear film lipids in dogs suffering from sebaceous adenitis was described recently (Striuli et al. 2024).

Significantly enlarged SGs and increased sebum production is observed in dogs with atopic dermatitis, probably causing the oily appearance of the coat in this disease (Wilkie et al. 1990). Morphological changes of SGs are also known in the context of otitis (van der Gaag 1986; Aslan et al. 2021). In the acute stage, in addition to infiltration with inflammatory cells, variable hyperplasia of the SGs with dilatation of the excretory ducts is often found in the skin of the ear canal (van der Gaag 1986; Huang et al. 2009; Aslan et al. 2021). Inflammation may also occur in the Meibomian gland as a chronic, painless protuberance (chalazion) on the conjunctiva. This leads to mechanical irritation, which can trigger conjunctivitis (Muller et al. 1993).

##### Neoplastic diseases

Tumors of the SGs, including sebaceous adenoma, sebaceous carcinoma, and sebaceous epithelioma, are frequently described in dogs (Moulton 1961; MacVean et al. 1978; Muller et al. 1993), accounting for 5 to 9% of all cutaneous neoplasias (Mukaratirwa et al. 2005; Kok et al. 2019; Martins et al. 2022). Frequently affected breeds are Cocker Spaniels, Kerry Blue Terriers, Boston Terriers, Poodles, Beagles, Dachshunds, Norwegian Elkhounds and Basset Hounds. The tumours are often located on the limbs or head including the eyelids (Krehbiel and Langham 1975; Strafuss 1976; Muller et al. 1993; Pakhrin et al. 2007; Kok et al. 2019; Baumgärtner and Gruber 2020; Martins et al. 2022). Adenomas are among the most frequent SG tumour types (Muller et al. 1993; Kok et al. 2019; Baumgärtner and Gruber 2020; Martins et al. 2022). They mainly contain mature sebocytes and are well demarcated from the surrounding tissue (Muller et al. 1993; Kim et al. 2024b). Adenomas can be distinguished from SG hyperplasia due to the presence of several layers of basal cells, in contrast to a single layer of basal cells. They can also affect the Meibomian gland (Albanese 2017). The ductal sebaceous adenoma, characterized by a proliferation of irregularly arranged ducts, is a special form of these tumors (Baumgärtner and Gruber 2020). Sebaceous carcinomas often reach a large size (Muller et al. 1993; Albanese 2017), but, despite their locally invasive growth, rarely form distant metastases (Baumgärtner and Gruber 2020). Sebaceous epitheliomas occur as frequently as adenoma of the SGs (Mukaratirwa et al. 2005; Chikweto et al. 2011) and resemble basal cell tumors of the epidermis. They consist largely of immature sebocytes (basal layer of SGs) with a high mitotic rate (Muller et al. 1993; Albanese 2017; Kim et al. 2024a).

Sebocytes can be present in tumors that do not primarily originate from SGs. Single reports describe mammary tumors in female dogs in which SG tissue was found (Chang et al. 2007; Grandi et al. 2011; Ma et al. 2024). To date, cases of SG differentiation have been found in invasive ductal carcinomas, in adenoid-cystic carcinomas and in intraductal adenomas with squamous metaplasia of the mammary glands (Grandi et al. 2011). Recently, a sebaceous carcinoma was described also within the lung of a dog (McDonald and Nakahara 2025).

##### Congenital diseases

Various congenital disorders in dogs are associated with impairment of the SGs. Hypohidrotic ectodermal dysplasia is a congenital defect that affects ectodermal structures such as skin glands, hair and teeth. Although these structures are already missing at the time of birth or are dysplastic, the animals are still viable. A breed predisposition has not been identified (Grieshaber et al. 1986; Moura et al. 2020). Other congenital disorders that can affect the function of SGs are ichthyosis, associated with enlarged SGs (Hoffmann et al. 2016), SG dysplasia, characterized by hypoplastic SGs formed by clusters of irregular basal cells and mature sebocytes (Peters-Kennedy et al. 2014), hamartomas which appears as a hairless, solitary protuberance consisting of large SG lobules in a random arrangement, each with a normal maturation pattern (Gross et al. 2005), and dermoid cysts, dermal to subcutaneous cavities that can open to the skin surface or be closed (Miller et al. 2012). Dermoid cysts usually occur in dogs along the dorsal midline (Saberi et al. 2013). The Rhodesian Ridgeback and Thai Ridgeback breeds appear to be predisposed to the formation of these cysts due to the typical ridge on their back (Mann and Stratton 1966; Hillbertz and Andersson 2006). A rather rare developmental malformation involving the SGs is the sebaceous nevus, which consists mainly of hyperplastic SG tissue, whereby HFs are usually underdeveloped and sweat glands are unchanged (Scarampella et al. 2000; Gross et al. 2005). They are often present from birth, but the exact mechanism of development has not been clarified (Muller et al. 1993; Scarampella et al. 2000).

Distichiasis in humans is a condition characterized by a partial or complete additional row of eyelashes (cilia). However, dogs normally have cilia only in the upper eyelid. Canine distichiasis, refers to cilia that arise in tarsal plate tissue, and emerge on the lid margin from the Meibomian gland openings, or less frequently from the Zeis or Moll gland openings. The glands themselves are histologically unchanged and fully functional (Raymond-Letron et al. 2012). Canine distichiasis may result from anomalous regulation of morphogenesis of HFs in the mesenchymal tissue of the tarsal plate.

##### Diseases of nutritional or toxicological origin

SG hyperplasia associated with hyperkeratosis and epidermal hyperplasia was found in Mexican hairless dogs suffering from contact hypersensitivity to stainless steel (Kimura 2007). A lack of essential FAs in the diet can also lead to long-term dermatological symptoms in dogs similar to those in cats (see above) (Muller et al. 1993).

##### Parasitic diseases

The SGs of dogs can harbor mites of the genus *Demodex*, in particular *D. canis* and, more rarely, *D. injai* (Desch and Hillier 2003; Ordeix et al. 2009; Baumgärtner and Gruber 2020; Deplazes et al. 2020). Massive multiplication of the parasites leads to dilatation of HFs and SGs as well as purulent folliculitis, especially in immunocompromised animals (Gruby 1846; Ferrer et al. 2014; Deplazes et al. 2020). In contrast to an infection with *D. canis*, greasy seborrhoea due to hyperplasia of the SGs can be observed during an infection with *D. injai* (Ordeix et al. 2009). Another parasitic disease involving the SGs is infection with the hookworm *Ancylostoma braziliense*. The larvae of these nematodes infect their hosts by penetrating the epidermis, passing through the SGs and leaving the dermis towards the subcutis. In humans, the larvae usually remain in the epidermis, possibly because of the significantly lower number of SGs in human skin. An infection pattern resembling that in humans occur on the footpads of dogs, where the SGs, necessary for invasion, are missing (Vetter and van der Linden 1977).

##### Diseases of unknown origin

As already mentioned, Mexican hairless dogs develop a skin disorder similar to acne vulgaris in humans. Comedones develop spontaneously on the back, limbs and prepuce of juvenile or adult animals (Kimura and Doi 1996). HFs are clogged with keratin and associated with well-developed SGs. Enlargement of these follicles to form dermal cysts is mainly observed in adult animals, although no inflammatory reaction is present. As these lesions progress, they may become solid cysts that appear macroscopically as raised areas of skin (Kimura and Doi 1996). Naked dogs with a higher sebum content on the skin developed comedones more frequently than those with a lower sebum content. The Mexican hairless dog is considered a suitable model for evaluating the efficacy of acne therapeutics, as their spontaneously occurring comedones are more similar to those of humans with acne than the chemically induced comedones of rabbits (Kimura and Doi 1996; Plewig and Kligman 2000). Topical treatment with common acne therapeutics used in human medicine resulted in the removal of keratin plugs in the affected naked dogs, but not in a quantitative reduction of comedones (Kimura and Doi 1996).

A clinical picture similar to acne is also known in normally haired dogs. Comedones and pustules are mainly found on the chin and lips and usually appear between three and twelve months of age, i.e., during adolescence, and in severe cases they persist into adulthood (Werner and Power 1994; Plewig and Kligman 2000; Shumaker 2020). Dogs with short, thick hair, such as Boxers, English Bulldogs, Doberman Pinschers and German and Danish Mastiffs are predisposed (Muller et al. 1993; Plewig and Kligman 2000). The lesions may be triggered by an increase in androgen levels during adolescence, causing hypertrophy and hyperplasia of the SGs, which in turn favor bacterial colonization and keratinization disorders of the follicular epithelium (Muller et al. 1993). In Miniature Schnauzer dogs, comedones usually first appear along the spine and then spread laterally; the disease is known as Schnauzer comedone syndrome (Muller et al. 1993).

Seborrhoea in dogs is a vaguely defined term that is used for various clinical pictures. It is most often characterized by increased sebum levels (Rosser et al. 1987), but dry seborrhoea, defined as excessive scaling of the skin including crusts or plaques triggered by a lack of sebum, also exists (Noli and Scarampella 2004). Seborrhoea usually occurs secondary to other diseases as SG nevus, malassezia dermatitis or a hormone-producing tumor (oily seborrhoea) or sebaceous adenitis, leishmaniasis, hyperadrenocorticism, cutaneous lymphoma, various autoimmune diseases or due to too frequent washing with degreasing shampoos (dry seborrhoea). Primary idiopathic seborrhoea is rare (Muller et al. 1993; Noli and Scarampella 2004; Baumgärtner and Gruber 2020; Shumaker 2020). Both forms include changes in the composition of the skin lipid film (Muller et al. 1993).

Border Terrier dogs have a predisposition to developing idiopathic generalized SG hyperplasia. The animals show a greasy coat with sticky hairs, and the SGs consist of a significantly higher number of lobules, while the size of the individual lobules is not changed (Dedola et al. 2010). However, the SG basal cells show significantly more mitoses compared to healthy animals. Other disorders of the SGs with an unknown cause are sebaceous retention cysts, presumably caused by blockage of the HF, which prevents the outflow of sebum (Muller et al. 1993), and discoid lupus erythematosus, often accompanied by atrophy of the HFs and associated SGs (Banovic et al. 2016).

Single cases of spontaneous SG hyperplasia, consisting of enlarged SG lobules arranged around a keratinized excretory duct, have been described (Okada 1992; Buendia et al. 2024). In old animals, this is known as senile nodular SG hyperplasia, non-neoplastic alterations occur that can be distinguished from papilloma by their greasy surface (Gross et al. 2005). Predisposed breeds include Poodles, Cocker Spaniels, Manchester Terriers and Wheaten Terriers (Gross et al. 2005).

## Summary

1. This review provides a comparative overview of the structure and function of SGs, the composition of sebum, and of SG-associated diseases in the species cattle, sheep, goat, pig, horse, cat and dog, the most common domestic and farm animals in the Western world.
2. The histological structure of the HF-associated SGs differs, if at all, only slightly between the species considered. In general, the body regions that have particularly large or numerous SGs are the mucocutaneous junctions, skin-horn junctions and the inguinal region. With regard to specialized SGs, the Meibomian gland and the free SGs of the external auditory canal exist in all species considered. Some animals also have specific free SGs, such as the intercornual and the preputial glands (goats), the inguinal glands and the interdigital glands (sheep), and the mental and circumoral organ (cats), to which a pheromone-producing function is often attributed.
3. In contrast to the SG structure, the composition of the sebum differs significantly between the species analyzed (Table 1). For example, the sebum of cats and cattle consists largely of type 1 wax diesters, whereas this lipid class does not occur in horses and dogs and only makes up a small proportion of sebum lipids in sheep. Equine sebum, in contrast, includes a high proportion (48%) of large-ring lactones, which are absent the sebum of other species.
4. These differences in sebum composition among the various species suggest functional differences. In horses, for example, thermoregulation through sweating plays a major role compared to the other species considered (Verdegaal et al. 2023). It therefore seems reasonable that horse sebum is optimized towards an emulsifying effect for sweat (Porter 2001). Cats, on the other hand, use the numerous SGs in the chin region and on the paws to mark their territory using scents (Cafazzo et al. 2019). Such marks should be retained as long as possible, which can be achieved with less volatile sebum components. In dogs, the skin glands of the circumanal region in particular are used for intra-species communication (Kokocińska-Kusiak et al. 2021); unlike in cats, as olfactory contact is made directly in dogs and no scent markings are left behind, the sebum does not have to be very volatile or persistent. In cattle, bulls seem to receive information about the stage of a cow’s sexual cycle from the secretions of the glands in the perineal region of female animals (Blazquez et al. 1987b). The high density of SGs in the udder skin can also make it easier for calves to find the udder (Ludewig 1996). It is generally assumed that the volatility of a molecule decreases with increasing molecular weight, but other properties such as polarity and the presence of functional groups also have an effect.
5. Fel d 1 is an important component of cat sebum, as it may trigger severe allergies in humans. Notably, the World Cat Federation lists a number of cat breeds as hypoallergenic (such as Balinese Cat, Cornish Rex, Devon Rex, Oriental Shorthair, Tiffanie Cat, Siberian Cat and Sphynx Cat), a correlation with sebum Fel d 1 levels in these breeds has not been examined (Satorina 2013).
6. Sebum production was demonstrated to be influenced by sex hormones, seasonality, the animal’s diet, and the breed. For example, an increase in SG volume was observed in thoroughbred horses in winter, while non-thoroughbreds had larger SGs in summer and no changes were observed in ponies (Sneddon et al. 2008). In cattle, ambient temperature and humidity have an influence on the amount and composition of sebum, which also differs between heat-tolerant and temperature-sensitive breeds (Smith et al. 1975; Poon et al. 1978; O'Kelly and Reich 1982). Seasonal changes in sebum amount and/or composition were also described in Saanen (Jenkinson et al. 1967; Sugiyama et al. 1986) and Turkish Angora goats (Sağlam et al. 1994).
7. A variety of pathologies can affect or involve the SGs in different mammalian species. Thus, SG tumors can occur in all species, even if dignity and typical localizations differ. With the exception of pigs, the infestation of SGs with Demodex mites has been documented in all species considered. Idiopathic inflammation of SGs is known in dogs, cats as well as horses and SG congenital malformations of have been observed in cattle, sheep, pigs, horses, cats, and dogs. Senile nodular SG hyperplasia is another disease known in both pets and humans.
8. The majority of studies and case reports on SG-related pathologies concern dogs and cats. This is presumably due to the fact that small animal medicine has reached a level that is comparable to human medicine in many fields and that non-life-threatening or performance-reducing pathologies such as dermatological diseases are more reliably recognized and treated in companion animals. In addition, dogs and cats in private ownership often reach an advanced age, which means that neoplastic diseases are detected more frequently.
9. There are hardly any reports of SG-associated diseases in pigs. A possible reason for this is the rudimentary character of the SG in pigs, whose loss of function is unlikely to have any significant effect on the skin health of the animals. Due to the low density and activity of the glands, which is supported by the poor enzyme activities, tumorous degeneration also seems less likely than in animals with a high density of very active SGs (Neurand and Meyer 1976). In addition, tumors of the SGs only occur in most species in middle to old age, which the majority of the pig population as slaughter animals usually does not reach.
10. A SG pathology similar to the acne vulgaris, one of the most common skin diseases in humans (van Steensel 2019), occurs in dogs and cats. While these animals may represent an interesting model organism for research into human acne, there are serious limitations, as the lesions in dogs and cats occur spontaneously and differ significantly from those in humans.

## Perspectives

As most of the original data on sebum lipid composition reported here was generated several decades ago, future studies employing state-of-the-art (spatial) lipidomic methods are likely to reveal so far unrecognized sebum components as well as their specific localization within the glands in different species. Formerly, the lipid analysis was mainly based on gas chromatography, which detects exclusively volatile compounds, and/or thin layer chromatography, which normally cannot unravel the fatty acyl residues in lipids. Nowadays, all this information can be obtained by modern mass spectrometry. Chromatography coupled to high resolution mass spectromtery and mass spectrometry imaging will give valuable insights into the molecular constellation of the skin and its glands. Therefore, this novel information will deliver valuable novel information about the structural (as the individual lipids and PL) and functional (such as antimicrobial activity or influence on ectoparasitic infestation) features of sebum. Such information may be of great practical value, for instance regarding ectoparasitic infestation. Understanding how sebum composition changes within life, the female cycle, following castration or during lactation may have the potential to develop different application methods or pharmaceutical components for diseased skin but also for anthelmintic drugs for beef cattle or dairy cows as well as for companion animals.

Knowledge about sebum composition and its regulation may open unexpected perspectives to improve livestock production and animal welfare, for instance regarding thermoregulation. Among livestock species, lactating dairy cows are particularly sensitive to high ambient temperatures because of their intense fermentative and metabolic heat production. Therefore, at high temperatures, lactating Holstein dairy cows decrease feed intake, milk yield, and milk constituent concentrations (Hut et al. 2022). As heat-resistant *Bos indicus* breeds were reported to possess higher amounts of sebaceous glands at different body regions compared to *Bos taurus* or crossbreed breeds (Mateescu et al. 2023; Naik et al. 2024), increased sebum secretion (besides increased sweat production) may improve heat loss via the skin, thus reducing heat stress. However, the regulation of sebum secretion and composition specifically in the mammary gland, a major site of heat flux (Gebremedhin and Wu 2016), and its role in heat dissipation at increased environmental temperatures have not been studied so far.

Another important development would be a better characterization of the skin microbiota in the different domestic animal species and their potential role in SG-related diseases, a topical research subject in human dermatology (Li and Jin 2025). While it is known that colonization by the commensal *S. aureus* over *C. acnes* may aggravate keratinocyte dysfunction and inflammation in human skin (Kreouzi et al. 2025), almost nothing is known about similar changes in animals. For instance, a comparable situation may be evoked by *S. pseudintermedius*, a coagulase-positive bacterium that shows significant microbiological similarities to *S. aureus* in humans and is particularly adept at colonizing the skin and mucous membranes of dogs (Wegener et al. 2018). Another microorganism that affects the secretion of SG, cause inflammation and is closely related to the pathogenesis of acne is *Malassezia*, a commensal lipophilic yeast of the human skin surface (Gaitanis et al. 2012). Malassezia are important skin commensals and opportunistic skin pathogens in a variety of animals (Guillot and Bond 2020), and particular relevant as the causative agent of dermatitis, otitis externa or paronychia in dogs and cats (Hobi et al. 2024).

As metabolic disorders cause skin lipid abnormalities in humans by influencing SG activity, future studies should address whether similar processes are related to skin diseases in domestic animals, such as dermatitis digitalis in cattle (Sölzer et al. 2022) or sweet itch in the horse, in particular in combination with the equine metabolic syndrome. The latter is known to be associated with alteration in serum insulin levels and increase of blood lipids (Frank et al. 2006; Durham et al. 2019), possibly leading to changes in sebum composition. Finally, studying sebaceous tumors, which are observed quite frequently in dogs, may be instructive for understanding tumor progress in humans. Thus, a more profound understanding of SG pathophysiology bears enormous potential to advance interdisciplinary research via cross-fertilization between human and veterinary medicine, and advance animal welfare and human health.

## Data Availability

Not applicable.

## Abbreviations

DGAT: Diacylglycerol acyltransferase
ELOVL5: Elongation of long-chain fatty acids family member 5
Fel d 1: Felis domesticus allergen 1
FFA: Free fatty acids
HF: Hair follicle
LRIG1: Leucine-rich repeats and immunoglobulin-like domains protein 1
PL: Phospholipids
SCD1: Stearoyl-CoA desaturase 1
SG: Sebaceous gland
SOAT1: Sterol O-acyltransferase 1
SOX9: SRY-box transcription factor 9
TAG: Triacylglycerols
WE: Wax esters
